# Prognostic impact of metformin in solid cancer patients receiving immune checkpoint inhibitors: novel evidences from a multicenter retrospective study

**DOI:** 10.3389/fphar.2024.1419498

**Published:** 2024-07-29

**Authors:** Jiaxin Wang, Jie Lin, Huaijuan Guo, Wenjuan Wu, Jingjing Yang, Jingxian Mao, Wenbin Fan, Hong Qiao, Ying Wang, Xuebing Yan, Hong Guo

**Affiliations:** ^1^ Department of Oncology, The Affiliated Hospital of Yangzhou University, Yangzhou University, Yangzhou, Jiangsu, China; ^2^ Department of Hepatobiliary and Pancreatic Surgery, Jilin University Second Hospital, Changchun, Jilin, China; ^3^ Department of Oncology, Northern Jiangsu People’s Hospital Affiliated to Yangzhou University, Yangzhou University, Yangzhou, Jiangsu, China; ^4^ Department of Oncology, Baoying Traditional Chinese Medicine Hospital, Yangzhou University, Yangzhou, Jiangsu, China; ^5^ Department of Thoracic Surgery, The Affiliated Hospital of Yangzhou University, Yangzhou University, Yangzhou, Jiangsu, China

**Keywords:** immune checkpoint inhibitor, cancer, metformin, prognosis, biomarker

## Abstract

**Objective:** Metformin as a common antidiabetic drug, has recently found to exert its anti-cancer and immunomodulatory effect in numerous preclinical studies. This study aims to clarify the prognostic impact of metformin use in solid cancer patients receiving immune checkpoint inhibitors (ICIs).

**Methods:** A retrospective cohort enrolling 516 solid cancer patients who received ICI-based therapy between 2018 and 2023 at three hospitals was analyzed. The primary endpoints included overall survival (OS) and progression-free survival (PFS). In addition, a bioinformatics analysis based on TCGA and GSE cohort was performed to investigate the prognostic significance of metformin target genes (MTGs) and their correlation with immune infiltration in non-small cell lung cancer (NSCLC) patients.

**Results:** In the entire cohort, a total of 76 patients received metformin before and/or during ICI therapy. The global analysis demonstrated that metformin use was unrelated with the OS (*p* = 0.064) and PFS (*p* = 0.059) of ICI-treated cancer patients, which was confirmed in the subgroups of esophagus, hepatobiliary or pancreatic cancer (all *p* > 0.05). However, metformin use was significantly correlated with better OS (*p* = 0.012) and PFS (*p* = 0.005) in ICI-treated lung cancer patients. Metformin use was also identified as an independent favorable prognostic factor for these patients. The bioinformatics analysis identified five favorable prognostic MTGs (RPS6KA5, RORA, SH3BP5, NUPR1, and CD40LG) for NSCLC patients, all of which was downregulated in lung cancer tissues as compared with normal tissues. The expressions of five MTGs not only could effectively stratify the OS of NSCLC patients, but also was correlated with infiltration of immune cells such as CD4^+^ and CD8^+^ T cells.

**Conclusion:** Metformin use was significantly correlated with better OS and PFS in ICI-treated lung cancer patients. MTGs has the potential to serve as novel clinical biomarkers or druggable targets for cancer immunotherapy. Considering study limitations, the actual impact of metformin use on ICI therapy needs to be clarified by more clinical trials.

## 1 Introduction

Cancer is one of major public health burdens worldwide, with approximately 20 million new cases and 9.7 million relevant deaths estimated in 2022 (Bray et al., 2024). Despite improved precancerous screening and diagnostic techniques, a considerable proportion of cancer patients are initially diagnosed at advanced stage and recommended to receive comprehensive therapies including surgery, chemoradiotherapy, targeted therapy and immunotherapy. The past decade has witnessed the great success of immunotherapy in advanced tumors and its representative drugs are known as immune checkpoint inhibitors (ICIs) including anti-programmed cell death-1 (PD-1), programmed cell death ligand-1 (PD-L1) and Cytotoxic T lymphocyte-associated antigen-4 (CTLA-4) antibodies (Capella et al., 2024; Jama et al., 2024). Although mounting clinical trials have proved their durable anti-cancer efficacy and acceptable toxicity, limited patients are actually found to benefit from ICI therapies. Numerous inherent factors have been closely linked to the efficacy of ICI drugs such as PD-L1 expression, microsatellite status, tumor mutation burden (TMB) and microbiome (Emens et al., 2024; Holder et al., 2024). Previously, our team has identified individual nutritional and performance status as significant factors affecting ICI efficacy (Yan et al., 2023; Wang et al., 2024). In addition, our team have found the actual efficacy of ICI drugs may be also affected by some concomitant medications including antibiotics, proton pump inhibitors, corticosteroids, β-blockers and opioids (Yang et al., 2020; Wang et al., 2021; Liu et al., 2022; Yan et al., 2022; Guo et al., 2024). A recent review has summarized the conflicting results about the impact of concomitant medications on ICI drugs in non-small cell lung cancer (NSCLC), emphasizing the necessity of more investigations on this aspect (Chen et al., 2023). A further understanding about the role of concomitant medications in cancer immunotherapy will undoubtedly contribute to more precise patient management, and finally lead to overall survival benefit.

Metformin, as a first-line medication for type 2 diabetes mellitus (T2DM), has recently exhibited its anti-cancer potential in numerous biological and clinical studies. A national cohort study has found melanoma patients with T2DM who received metformin had reduced risk of cancer-specific mortality (Urbonas et al., 2020). A comprehensive meta-analysis including 166 studies has proved metformin use is significantly correlated with a decreased risk for gastrointestinal, urologic and hematologic cancers (O’connor et al., 2024). Metformin use is also correlated with better clinical outcome in cancer patients and can act as an effective adjuvant therapy combined with traditional chemoradiotherapy (Júnior et al., 2021; Bahardoust et al., 2024). In terms of anti-cancer mechanisms, metformin can directly inhibit the malignant characteristics of cancer cells through activating AMPK signaling, or indirectly prevent tumorigenesis through controlling circulating glucose and insulin levels (Linkeviciute-Ulinskiene et al., 2020). A recent review has closely linked metformin use with increased CD8^+^ T cells and natural killer (NK) cells, suggesting its potential boosting effect on cancer immunotherapy (Panaampon et al., 2023). However, the actual impact of metformin use on ICI efficacy remains controversial in clinical studies. For instance, Afzal et al. have found ICIs combined with metformin could effectively improve the tumor response, overall survival (OS) and progression-free survival (PFS) of NSCLC patients (Afzal et al., 2019). In contrast, another retrospective study has demonstrated no significant correlation between metformin use and clinical outcome in NSCLC patients receiving nivolumab (Svaton et al., 2020). Moreover, a multicenter retrospective study even has found metformin use was correlated with increased risk of disease progression and death in ICI-treated solid cancer patients (Cortellini et al., 2023). Therefore, more investigations are urgently needed to clarify the actual role of metformin in cancer immunotherapy.

In this study, a multicenter cohort of 516 solid cancer patients receiving ICI-based therapies was used to evaluate the impact of metformin use on patient prognosis. In addition, a comprehensive bioinformatic analysis was performed to investigate the potential correlation between the metformin target genes (MTGs) and immune cells. The study will provide novel insights into the anti-cancer role of metformin, contributing to precise management of concomitant medications during ICI therapy.

## 2 Materials and methods

### 2.1 Study design and patient information

**FIGURE 1:**
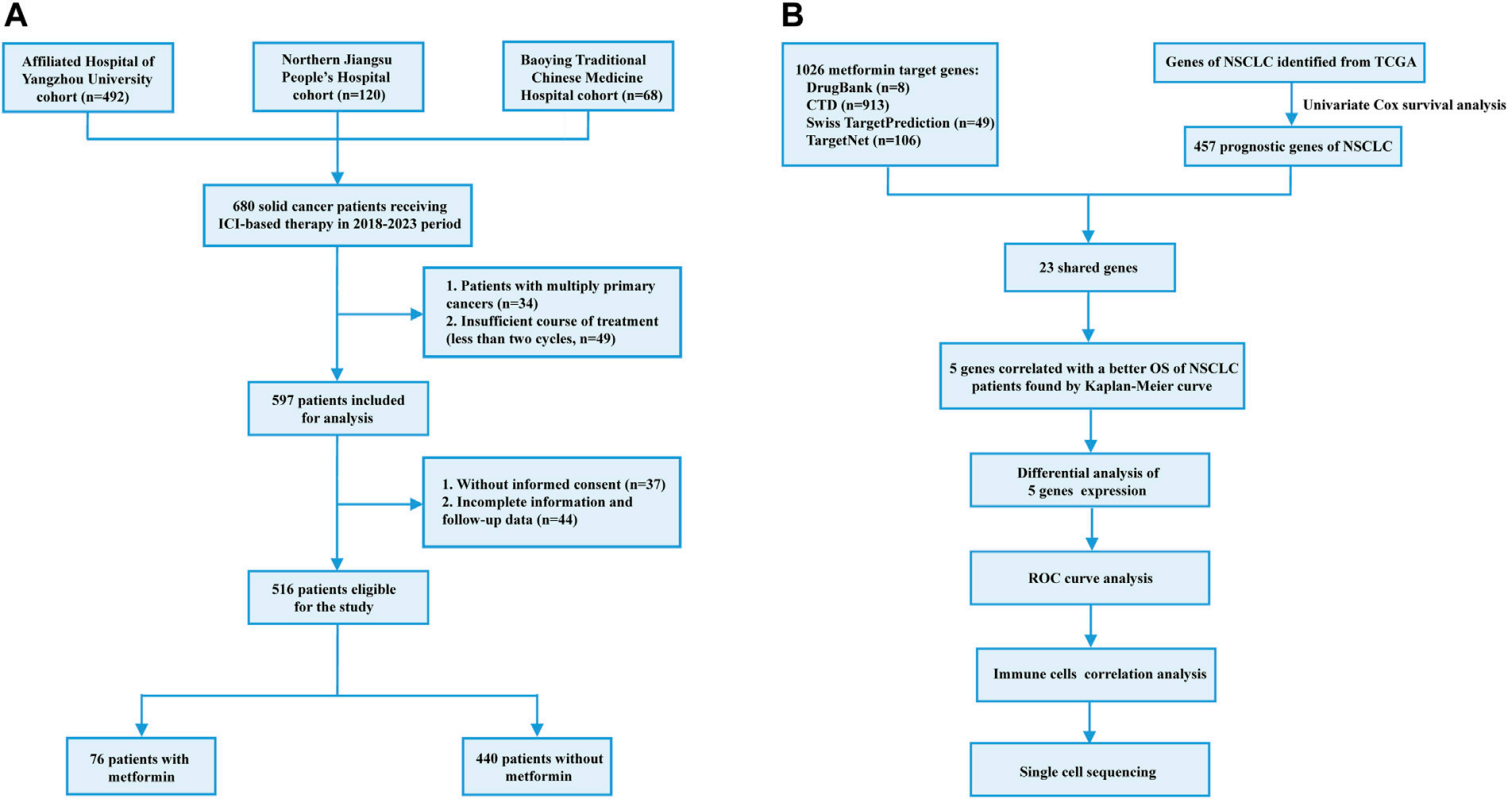
Flowchart of patient recruitment in the retrospective study **(A)** and identification of metformin target genes **(B)**.

Between January 2018 and December 2023, a total of 680 patients were initially selected from three medical centers: The Affiliated Hospital of Yangzhou University (n = 492), Northern Jiangsu People’s Hospital Affiliated to Yangzhou University (n = 120) and Baoying Traditional Chinese Medicine Hospital (n = 68). The inclusion criteria were as follows: 1) age over 18 years old; 2) patients were pathologically diagnosed as solid cancers including lung and digestive cancers; 3) patients received ICI therapy with or without other anti-cancer therapies including chemotherapy, radiotherapy and targeted therapy. The exclusion criteria were as follows: 1) multiple primary tumors; 2) incomplete medical and/or follow-up records; 3) insufficient ICI therapy (less than two cycles); 4) unavailable informed consents for using patient information. As a result, a total of 516 patients were included in the study, among which 76 patients received metformin therapy before and/or during ICI therapy. The flowchart of patient recruitment was shown in Figure 1A. This study was approved by the local ethics committee (No. 2022-YKL11-class 05) and informed consents were acquired from patients or their legal guardians for using their medical and follow-up records in scientific researches.

### 2.2 Treatment strategy

All the included patients received ICI therapy every two or three weeks. The types of ICI drugs were as follows: sintilimab (n = 184), camrelizumab (n = 134), tirelizumab (n = 108), toripalimab (n = 22), pembrolizumab (n = 20), serplulimab (n = 17), nivolumab (n = 11), durvalumab (n = 7), envafolimab (n = 6), atezolizumab (n = 4), penpulimab (n = 2) and adebrelimab (n = 1). 447 and 33 patients received chemotherapy and radiotherapy respectively. 106 patients received targeted therapy and the used drugs were as follows: anlotinib (n = 26), apatinib (n = 23), lenvatinib (n = 20), bevacizumab (n = 16), trastuzumab (n = 8), regorafenib (n = 5), sulfatinib (n = 3), sorafenib (n = 2), pyrotinib (n = 1), furoquinib (n = 1), gefitinib (n = 1) and nimotuzumab (n = 1).

### 2.3 Follow-up and study endpoints

For oncological evaluation, all the included patients received tumor marker detection and radiological examination every two or three cycles. The anti-cancer therapy response was evaluated based on the Response Evaluation Criteria in Solid Tumors (RECIST) 1.1. The therapy decision was performed based on oncological and safety evaluation. The study endpoints contained OS and PFS. OS was defined as the time interval between the first ICI therapy and death from any cause or the last follow-up. PFS was defined as the time interval between the first ICI therapy and disease progression.

### 2.4 Identification of prognostic metformin target genes in online databases

The metformin target genes (MTGs) were obtained from the DrugBank (Knox et al., 2024) (https://go.drugbank.com), Comparative Toxicogenomics Database (Davis et al., 2023) (https://ctdbase.org/), Swiss Target Prediction (Daina et al., 2019) (http://www.swisstargetprediction.ch/) and TargetNet (Yao et al., 2016) (http://targetnet.scbdd.com/calcnet/index/). In addition, the transcriptomic data of Lung Adenocarcinoma (LUAD) and Lung Squamous Cell Carcinoma (LUSC) from The Cancer Genome Atlas (TCGA, https://portal.gdc.cancer.gov/v1/) were downloaded as the NSCLC dataset. Firstly, favorable prognostic genes were identified from the NSCLC dataset using the univariate Cox regression method. The eligibility criteria were used as follows: 1) *p*-value less than 0.05; 2) hazard ratio (HR) value less than 1. Secondly, the shared genes of both the favorable NSCLC prognostic genes and MTGs were selected. Finally, the Kaplan-Meier model was utilized to validate the prognostic value of the prognostic MTGs in the NSCLC dataset. The flowchart of identifying prognostic MTGs was shown in Figure 1B.

### 2.5 Immune infiltration analysis

The correlation between MTGs and proportions of immune cells was analyzed using single sample gene set enrichment analysis (ssGSEA) in “GSVA” and “GSEABase” packages. In addition, the expressions of MTGs in immune cells of NSCLC patients were analyzed using single-cell sequencing data that were available in TISCH database (http://tisch.comp-genomics.org/home/).

### 2.6 Statistical analysis

The statistical analysis was performed using SPSS 25.0 or R 4.3.0 software. The correlations between metformin use and clinical features were analyzed using chi-squared test. The survival curves were plotted using Kaplan-Meier model and intergroup difference was evaluated using the log-rank test. Independent prognostic factors were identified using the univariate and multivariate analysis based on Cox proportional hazards regression model. The performance of MTGs in predicting clinical outcome was analyzed using receiver operator characteristic (ROC) curves. A *p*-value less than 0.05 indicates statistical significance.

## 3 Results

### 3.1 General description of patient characteristics in the multicenter cohort

Based on the inclusion and exclusion criteria, a total of 516 patients were finally selected for our retrospective analysis and their clinical features were shown in Table 1. In brief, 123 (23.8%) and 393 (76.2%) patients were female and male respectively, with the overall median age of 68 years old. The most common cancer type was lung cancer (n = 199), followed by esophageal cancer (n = 164), gastrointestinal cancer (n = 85), hepatobiliary and pancreatic cancer (n = 68). 125 (24.2%) patients had previously received tumor resection and 209 (40.5%) patients had smoking history. Only 36 (7.0%) patients received ICI monotherapy, while the others received combined therapies. Before the last follow-up, 294 patients were dead from tumor progression while 55 patients were dead from other reasons such as infection, cerebrovascular diseases and therapy-related adverse events. 76 patients received metformin therapy with drug dose ranging from 500 mg to 2000 mg per day. The correlation analysis demonstrated metformin use were significantly correlated with body mass index (BMI) (*p* = 0.028) and treatment strategy (*p* = 0.005). No significant correlation was observed between metformin use and other clinical features including gender (*p* = 0.973), age (*p* = 0.955), cancer type (*p* = 0.231), surgery history (*p* = 0.453), tumor staging (*p* = 0.333), ECOG score (*p* = 0.568) and smoking history (*p* = 0.286).

**TABLE 1:** Baseline characteristics of the entire cohort.

Clinical features	Total	Non-metformin (n = 440)	Metformin (n = 76)	*p*-value
Gender				0.973
Female	123	105	18	
Male	393	335	58	
Age				0.955
<65	178	152	26	
≥65	338	288	50	
Cancer				0.231
Lung	199	165	34	
Digestive system	317	275	42	
Esophageal	164	142	22	
Gastrointestinal	85	76	9	
Hepatobiliary and Pancreatic	68	57	11	
Surgery history				0.453
No	391	336	55	
Yes	125	104	21	
Clinical Tumor Staging				0.333
III	133	110	23	
IV	383	330	53	
ECOG				0.568
0–1	426	365	61	
≥2	90	75	15	
Smoking				0.286
Never	307	266	41	
Current/former	209	174	35	
Treatment				0.005
Monotherapy	36	25	11	
Combination	480	415	65	
BMI				0.028
<28 kg/m^2^	310	273	37	
≥28 kg/m^2^	206	167	39	

Abbreviations: ECOG, eastern cooperative oncology group; BMI, body mass index.

### 3.2 Prognostic significance of metformin use in the multicenter cohort

As shown in Supplemenary Figure S1A, no statistically significant difference was observed in the OS between the metformin group and non-metformin group (*p* = 0.064). Similarly, the PFS of the metformin group was found to be no better than that of non-metformin group (*p* = 0.059, Supplemenary Figure S1B). In addition, the univariate analysis failed to identify metformin use as a significant prognostic factor affecting the OS or PFS of the patients (OS: *p* = 0.073; PFS: *p* = 0.072; Table 2).

**TABLE 2:** Univariate analysis for overall survival and progression-free survival of the entire cohort.

Characteristics	Univariate analysis					
	Overall survival			Progression-free survival		
	HR	95% CI	p	HR	95% CI	p
Gender	0.876	0.684–1.123	0.297	0.891	0.703–1.130	0.341
Age	1.243	0.994–1.554	0.056	1.170	0.949–1.442	0.141
Cancer	1.371	1.104–1.702	0.004	1.431	1.164–1.758	0.001
Surgery history	0.784	0.607–1.013	0.062	0.957	0.758–1.207	0.709
Staging	1.277	0.999–1.633	0.051	1.456	1.151–1.843	0.002
ECOG	1.744	1.353–2.248	<0.001	1.799	1.410–2.296	<0.001
BMI	0.825	0.664–1.025	0.082	0.896	0.731–1.099	0.291
Smoking	1.009	0.817–1.246	0.933	0.978	0.800–1.194	0.824
Treatment	0.887	0.581–1.356	0.581	1.364	0.887–2.098	0.158
Metformin	0.759	0.562–1.026	0.073	0.774	0.585–1.024	0.072

Abbreviations: ECOG, eastern cooperative oncology group; HR, hazard ratio; 95%CI, confidence interval; BMI, body mass index.

### 3.3 Prognostic significance of metformin use in the selected cancer types

**FIGURE 2:**
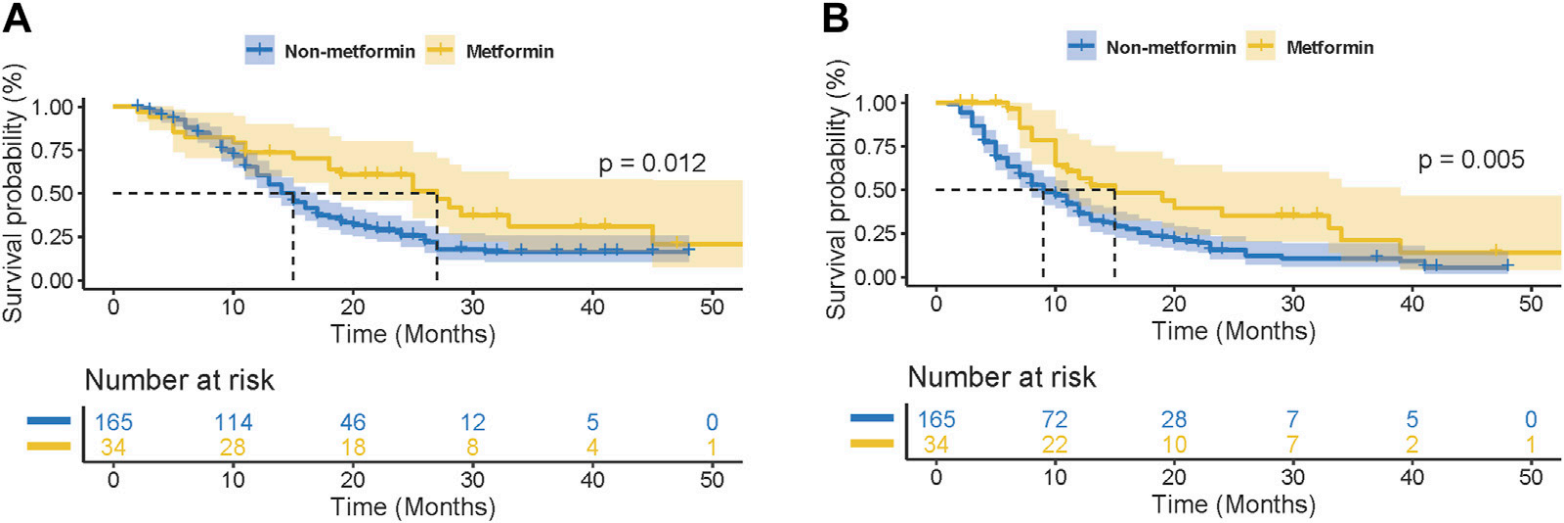
Kaplan-Meier curves for the association of metformin use with overall survival (OS) **(A)** and progression-free survival (PFS) **(B)** in lung cancer patients receiving immune checkpoint inhibitors.

For further clarifying the prognostic significance of metformin use in ICI-treated patients, the subgroup analysis was performed based on cancer types. In patients with lung cancer (n = 199), 34 patients received metformin therapy. As shown in Figures 2A,B, metformin use was found to be significantly correlated with better OS and PFS in ICI-treated lung cancer patients (OS: *p* = 0.012; PFS: *p* = 0.005). This correlation was also statistically significant in the subgroup analysis based on small cell lung cancer (SCLC, OS: *p* = 0.010, Supplementary Figure S2A; PFS: *p* = 0.017; Supplementary Figure S2B) and NSCLC (OS: *p* = 0.033, Supplementary Figure S2C; PFS: *p* = 0.009; Supplementary Figure S2D). In the univariate analysis, metformin use (*p* = 0.008), together with tumor staging (*p* = 0.005), ECOG (*p* = 0.001) and smoking history (*p* = 0.005) were significant factors affecting the PFS of lung cancer patients (Table 3). In the multivariate analysis, metformin use (*p* = 0.013), together with tumor staging (*p* = 0.017) and smoking history (*p* = 0.028) were further identified as independent predictive factors for the PFS of lung cancer patients. In terms of OS, metformin use (*p* = 0.016), ECOG (*p* = 0.016) and smoking history (*p* = 0.023) were significant prognostic factors in the univariate analysis (Table 4). However, only metformin use was found to be an independent predictive factor for OS (*p* = 0.026).

**TABLE 3:** Univariate and multivariate analysis for the progression-free survival of the lung cancer cohort.

Characteristics	Progression-free survival					
	Univariate analysis			Multivariate analysis		
	HR	95% CI	p	HR	95% CI	p
Gender	1.014	0.627–1.641	0.954			
Age	1.225	0.857–1.752	0.265			
Cancer	0.665	0.406–1.091	0.106			
Surgery history	0.791	0.515–1.216	0.286			
Tumor staging	1.715	1.174–2.505	0.005	1.596	1.086–2.345	0.017
ECOG	1.920	1.284–2.871	0.001	1.481	0.976–2.250	0.065
BMI	0.814	0.586–1.129	0.218			
Smoking	1.771	1.194–2.628	0.005	1.575	1.051–2.361	0.028
Treatment	1.476	0.775–2.810	0.236			
Metformin	0.527	0.328–0.846	0.008	0.547	0.340–0.881	0.013

Abbreviations: ECOG, eastern cooperative oncology group; HR, hazard ratio; 95%CI, confidence interval; BMI, body mass index.

**TABLE 4:** Univariate and multivariate analysis for the overall survival of the lung cancer cohort.

Characteristics	Overall survival					
	Univariate analysis			Multivariate analysis		
	HR	95% CI	p	HR	95% CI	p
Gender	0.828	0.515–1.330	0.435			
Age	1.155	0.798–1.671	0.445			
Cancer	0.796	0.478–1.325	0.380			
Surgery history	0.727	0.456–1.158	1.179			
Tumor staging	1.387	0.941–2.045	0.098			
ECOG	1.673	1.103–2.540	0.016	1.427	0.929–2.191	0.104
BMI	0.770	0.542–1.093	0.143			
Smoking	1.670	1.074–2.596	0.023	1.559	0.993–2.445	0.053
Treatment	0.952	0.512–1.770	0.876			
Metformin	0.553	0.342–0.894	0.016	0.577	0.356–0.937	0.026

Abbreviations: ECOG, eastern cooperative oncology group; HR, hazard ratio; 95%CI, confidence interval; BMI, body mass index.

In patients with esophagus cancer (n = 164), 22 patients received metformin therapy. As shown in Supplementary Figure S3A and B, no significant correlation was observed between metformin use and OS (*p* = 0.672) or PFS (*p* = 0.898). In patients with hepatobiliary or pancreatic cancer (n = 68), 11 patients received metformin therapy. The correlation between metformin use and clinical outcome still failed to be statistically significant (OS: *p* = 0.439, Supplementary Figure S3C; PFS: *p* = 0.754; Supplementary Figure S3D). We failed to perform the analysis in the gastrointestinal subgroup due to the limited sample size of the metformin group (n = 9).

### 3.4 Identification of favorable prognostic MTGs in NSCLC patients

**FIGURE 3:**
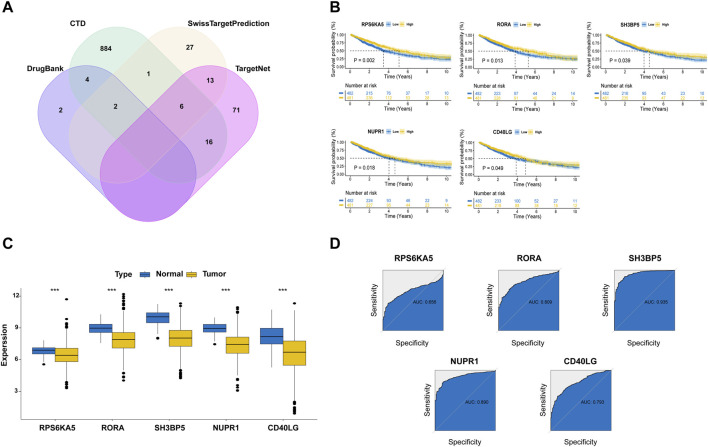
Identification of prognostic metformin target genes (MTGs) in NSCLC patients. **(A)** Veen plot for the MTGs in the online databases. **(B)** The survival curves stratified by expressions of five MTGs in NSCLC patients from TCGA cohort. **(C)** Expressions of five MTGs in the tumor and normal tissues of NSCLC patients from TCGA cohort. **(D)** Receiver operating characteristic curves for determining the predictive performance of five MTGs in predicting the OS of NSCLC patients from TCGA cohort.

Since our subgroup analysis revealed metformin use was associated with better clinical outcome in ICI-treated lung cancer patients, we next aimed to investigate the underlying molecular mechanisms based on network pharmacology. As shown in Figure 3A, a total of 1,026 MTGs were initially identified from four online databases and the details were provided in Supplementary Table S1. Meanwhile, using a univariate Cox regression model, 457 favorable prognostic genes related with NSCLC were identified from the TCGA cohort and the details were provided in Supplementary Table S2. Then, the following 23 shared genes between MTGs and favorable prognostic genes were selected: SLC47A1, CYP17A1, RPS6KA5, TP53INP1, ABCC4, BCL6, CCR2, CD40LG, CD74, CISH, GDF15, GMPR, IL33, MCTP2, NUPR1, PLEKHB1, PLPPR1, PXMP4, SH3BP5, TLR2, TLR5, CA5B, and RORA. The Kaplan-Meier model was used to validate the prognostic significance of these genes in the NSCLC cohort. As a result, five MTGs (RPS6KA5, RORA, SH3BP5, NUPR1 and CD40LG) were found to be significantly correlated with a better OS of NSCLC patients (Figure 3B). The further analysis demonstrated the expressions of these five MTGs were all significantly downregulated in tumor tissues as compared with those in normal tissues in NSCLC patients (Figure 3C). Finally, the ROC analysis was used to evaluate their performance in predicting the OS of NSCLC patients (Figure 3D). The result demonstrated SH3BP5 had the best predictive performance with AUC of 0.935, followed by NUPR1 (AUC = 0.890), RORA (AUC = 0.809), CD40LG (AUC = 0.793) and RPS6KA5 (AUC = 0.656).

### 3.5 Correlation of favorable prognostic MTGs with immune infiltration in NSCLC patients

**FIGURE 4:**
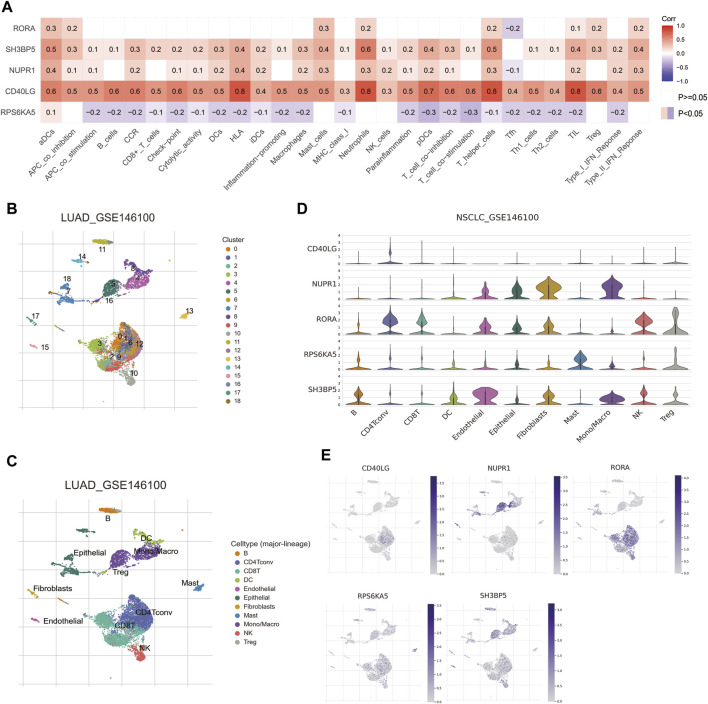
Correlations of prognostic metformin target genes (MTGs) with immune infiltration in NSCLC patients. **(A)** Correlations of five prognostic MTGs with immune infiltration in NSCLC patients from TCGA cohort. **(B and C)** Cell distribution **(B)** and matched annotation **(C)** of NSCLC patients from GSE146100 cohort. **(D)** Expression profiles of five prognostic MTGs in various immune cells. **(E)** Localization of five prognostic MTGs in immune cells.

As shown in Figure 4A, the ssGSEA analysis indicated the expressions of four favorable prognostic MTGs (RORA, SH3BP5, NUPR1 and CD40LG) were positively correlated with proportion of most infiltrated immune cells. For example, CD40LG expression was positively correlated with the proportion of activated dendritic cells, B cells, CD8^+^ T cells, check-point, macrophage, etc. For further investigating the cellular distribution of these MTGs, a NSCLC single-cell dataset was utilized (GSE146100). The distributions of cell types and matched annotations were demonstrated in Figures 4B,C respectively. The relative quantitative analysis for detecting gene expressions in immune cells was then performed and the result was shown in Figure 4D. For example, the expression of RORA was significantly increased in CD4^+^ T cells, CD8^+^ T cells and natural killer cells. The expression of NUPR1 was significantly increased in monocytes or macrophages, while that of SH3BP5 was abundant in B cells, natural killer cells and Treg cells. Finally, the cellular localization analysis confirmed the correlation between MTGs expression and immune cells (Figure 4E).

## 4 Discussion

Despite encouraging results from biological experiments, the actual efficacy of metformin in combination with anti-cancer therapies is still controversial. In locally advanced NSCLC patients, additional use of metformin resulted in worse clinical outcome and increased toxic events (Tsakiridis et al., 2021). A retrospective analysis found metformin use failed to provide long-term survival benefit in colorectal cancer patients receiving neoadjuvant therapy followed by surgical resection (Sonal et al., 2024). A Phase I/II trial (NCT02949700) is ongoing to identify metformin as a chemo-radiosensitizer for head and neck cancer patients (Kemnade et al., 2023). The preliminary result has demonstrated an improving trend of patient survival in the metformin group, although it failed to reach statistical significance. To our knowledge, a recent meta-analysis including 22 studies has summarized the retrospective studies regarding the role of metformin use in combination with ICI therapy (Shen et al., 2024). The result suggests metformin use is significantly correlated with worse OS (*p* = 0.004) instead of PFS (*p* = 0.345) in ICI-treated cancer patients. However, various inherent factors such as patient selection and therapy strategies may result in the heterogeneous results of the meta-analysis. Therefore, more clinical investigations with sufficient sample sizes are urgently needed. In this study, using a multicenter cohort, we found metformin use was significantly correlated with better outcome in ICI-treated lung cancer patients instead of other cancer patients, which may be partly attributed to the role of its target genes in activating immune cells. This finding provides novel evidences for the utilization of metformin as a promising adjuvant drug in cancer immunotherapy.

For the entire cohort, no significant correlation was observed between metformin use and clinical outcome. This finding is consistent with several published retrospective studies (Buti et al., 2021; Gaucher et al., 2021). A recent large-scale multicenter study (n = 1,395) has even found metformin use was associated with increased risk of disease progression and death in ICI-treated patients with advanced solid cancers (Cortellini et al., 2023). The researchers speculated that metformin use may impair the anti-cancer immune system through affecting gut microbiome or immune related cytokines. On the other hand, another multicenter study (n = 878) has demonstrated that concomitant use of metformin was associated with better clinical outcome in ICI-treated cancer patients, while this beneficial effect was not observed in patients who only received metformin before ICI therapy (Chiang et al., 2023). In clinical practice, number prognostic factors vary greatly among different cancers, such as pathological types, therapeutic strategies and immune microenvironment. Therefore, our global analysis may be insufficient to accurately evaluate the correlation between metformin use and ICI efficacy, suggesting the necessity of subgroup analysis.

In our subgroup of lung cancer, the survival analysis demonstrated that metformin use was significantly correlated with better OS and PFS in ICI treated patients. The univariate and multivariate analysis identified metformin use was an independent favorable prognostic factor. These findings collectively supported the beneficial role of metformin in combination with ICI drugs, which was consistent with several published studies. For instance, Afzal et al. found metformin use was correlated with better disease control and response rate in NSCLC patients receiving ICIs as second or third-line therapy (Afzal et al., 2019). Similarly, Yang et al. found the use of metformin with or without dipeptidyl peptidase four inhibitors was correlated with higher objective response rate and longer PFS in metastatic NSCLC patients who received ICI monotherapy (Yang et al., 2023). A published case report demonstrated metformin has the potential to overcome acquired resistance to nivolumab in small cell lung cancer patients (Kim et al., 2021). Some recent mechanism investigations can be used for explaining the beneficial role of metformin use in ICI-treated patients with lung cancer. In lung cancer bearing mice, metformin increased CD8^+^ T cell infiltration and IFN-γ expression through modulating gut microbiota, contributing to enhanced anti-cancer immunity (Zhao et al., 2024). Metformin was found to promote the formation of memory CD8^+^ T cells and inhibit their apoptosis, enabling increased tumor-infiltrating CD8^+^ T cells in lung cancer patients (Zhang et al., 2020). Metformin could even directly decreased the expressions of both PD-1 and PD-L1, creating a favorable microenvironment to prevent tumor immune evasion (Park et al., 2024). It should be noted that two studies failed to demonstrate its beneficial role in ICI-treated lung cancer patients, which may be partly attributed to the potential impact of confounding factors (such as corticosteroids, antibiotics, proton pump inhibitors, etc.) in the multivariate analysis (Svaton et al., 2020; Cortellini et al., 2021).

In the subgroup analysis for esophagus cancer patients, no significant correlation was observed between metformin use and clinical outcome. This result was inconsistent with a mechanism investigation that found metformin improved the immunosuppressive tumor microenvironment in an esophageal spontaneous carcinogenesis rat model (Takei et al., 2022). Although previous studies have confirmed the preventive role of metformin use in esophageal carcinogenesis, relevant clinical evidences for its correlation with ICI drugs are lacking and further efforts are needed (Najafi et al., 2023). With regard to patients with hepatobiliary or pancreatic cancer, the similar result was observed. A recent retrospective study has even found metformin use was associated with worse objective response, median OS and PFS in ICI-treated patients with advanced hepatocellular carcinoma (Kang et al., 2023). This finding was contradictory with a recent comprehensive review that highlighted its role in improving immune microenvironment and regulating expressions of immune genes in hepatocellular carcinoma (Abd El-Fattah and Zakaria, 2022). In pancreatic cancer patients who received gemcitabine-based neoadjuvant chemoradiotherapy, metformin use could reduce pro-tumoral M2 macrophages and increase immune-activating dendritic cells, further supporting its beneficial role in immunotherapy (van Eijck et al., 2024). Considering the great differences between preclinical experiments and clinical studies, more well-designed clinical trials are urgently needed for further validation.

Since we found metformin use was correlated with better clinical outcome in ICI-treated lung cancer patients, we next made efforts to investigate the underlying mechanisms based on the bioinformatics method. As result, we identified five target genes of metformin (RPS6KA5, RORA, SH3BP5, NUPR1, and CD40LG), which were significantly correlated with favorable prognosis and immune infiltration in lung cancer patients. To our knowledge, some recently published studies have correlated these genes with lung cancer. RPS6KA5, as a substrate of MAPK activated protein kinase family, was found to induce humoral immune response and its autoantibody could be used to diagnosis lung cancer (Pei et al., 2020). High RORA expression was proved as an independent favorable factor for OS and correlated with numerous immune checkpoint-related genes such as CD274 and PDCD1LG2 in NSCLC patients (Xian et al., 2022). SH3BP5 was identified as a downstream target of METTL3 that inhibited lung cancer invasion through regulating SH3BP5 mRNA stability in a YTHDF1-dependent manner (Zhang et al., 2024). Metformin upregulated NUPR1 expression in NSCLC cells, while knockdown of NUPR1 induced cell sensitivity to metformin or ionizing radiation (Kim et al., 2022). CD40LG not only could promote the apoptosis of lung cancer cells, but also may be involved in regulating T cell function (Xu et al., 2010; Guo et al., 2023). Although direct clinical evidences are lacking, these MTGs have the potential to be developed as novel clinical biomarkers for ICI-treated lung cancer patients.

Our retrospective study has some inherent limitations. Firstly, the proportion of patients who received metformin was relatively small (76/516, 14.7%), which hampers further subgroup analysis. Therefore, multicenter validations based on larger sample size are essential. Secondly, due to the retrospective nature, numerous heterogeneous factors such as patient selection, cancer type, ICI drug and metformin doses significantly affect the results. For overcoming this limitation, more randomized controlled trials with rigorous design are highly encouraged. Thirdly, we failed to assess the impact of the cumulative effect of metformin doses, duration of DM and other antidiabetic drugs, all of which should be emphasized in our following work. Finally, the bioinformatics method was used to identify prognostic MTGs and clarify their correlations with immune cells, which needs further verification based on clinical samples and biological experiments.

In conclusion, metformin use was significantly correlated with better OS and PFS in ICI-treated lung cancer patients. In addition, five MTGs were identified as prognostic biomarkers for lung cancer patients, which was correlated with infiltration of immune cells. The actual role of metformin and its target genes in cancer immunotherapy still need to be clarified by more work in future.

## Data Availability

The original contributions presented in the study are included in the article/Supplementary Material, further inquiries can be directed to the corresponding authors.

## Supplementary material

SUPPLEMENTARY FIGURE S1 Kaplan-Meier curves for the association of metformin use with OS **(A)** and PFS **(B)** in solid cancer patients receiving immune checkpoint inhibitors.

SUPPLEMENTARY FIGURE S2 Prognostic significance of metformin use in the subgroup analysis based on small and non-small cell lung cancer. **(A, B)** Kaplan-Meier curves for the association of metformin use with OS **(A)** and PFS **(B)** in small cell lung cancer patients receiving immune checkpoint inhibitors (ICIs). **(C, D)** Kaplan-Meier curves for the association of metformin use with OS **(C)** and PFS **(D**) in non-small cell lung cancer patients receiving ICIs.

SUPPLEMENTARY FIGURE S3 Prognostic significance of metformin use in the subgroup analysis based on cancer types. **(A, B**) Kaplan-Meier curves for the association of metformin use with OS **(A)** and PFS (B) in esophagus cancer patients receiving ICIs. **(C, D)** Kaplan-Meier curves for the association of metformin use with OS **(C)** and PFS **(D)** in hepatobiliary and pancreatic cancer patients receiving ICIs.
