# Supplementary material for: Prognostic impact of metformin in solid cancer patients receiving immune checkpoint inhibitors: novel evidences from a multicenter retrospective study
Source: Front Pharmacol. 2024 Jul 29;15:1419498. doi: 10.3389/fphar.2024.1419498 (PMC11317293; doi:10.3389/fphar.2024.1419498)
Supplement: Supplementary file 1 [file Table1.DOCX]

Supplementary Table 1. Metformin target genes identified from online databases

| CTD | SWISS | TargetNet | Drugbank |
| --- | --- | --- | --- |
| INS | XDH | PRSS2 | SLC22A2 |
| TNF | PLAU | NOS1 | SLC47A1 |
| PRKAA1 | PLAUR | NOS2 | ETFDH |
| AKT1 | UPP1 | NOS3 | PRKAB1 |
| CASP3 | PRSS1 | SLC9A1 | SLC22A1 |
| IL1B | TAAR1 | HTR1E | SLC29A4 |
| IL13 | TYMS | ADRA2C | SLC22A3 |
| IL6 | TYMP | CA5B | SLC47A2 |
| SLC2A4 | F2 | CA4 |  |
| PPARGC1A | SLC22A1 | CA6 |  |
| SLC22A2 | HTR5A | CHRM4 |  |
| SLC47A1 | S100B | S1PR2 |  |
| SIRT3 | PNP | CA7 |  |
| CAT | PLG | CHRNA4 |  |
| ACACA | PDE5A | CA5A |  |
| HMOX1 | NOS3 | CA12 |  |
| NFE2L2 | NOS1 | CES2 |  |
| PRKAA2 | NOS2 | PLAT |  |
| RELA | NISCH | ALOX5 |  |
| SIRT1 | CHRNB2 | ALPL |  |
| BAX | CHRNA4 | MAOB |  |
| FSHB | SLC47A1 | APOBEC3A |  |
| GSK3B | METAP2 | MAOA |  |
| NQO1 | KLK1 | TLR9 |  |
| BCL2 | ITGAV | ALOX15 |  |
| IGF1 | INMT | CA13 |  |
| PARP1 | IDO1 | MGLL |  |
| PPARG | HRH4 | CASP9 |  |
| BMP2 | HRH3 | FDPS |  |
| CYP1A1 | HRH2 | RELA |  |
| INS1 | GDA | BCL2A1 |  |
| SLC19A3 | GPR84 | ACHE |  |
| SLC22A3 | ESR2 | HTR5A |  |
| ABCB1 | EGFR | CA14 |  |
| CCL2 | DPYD | CHRM2 |  |
| CYP3A4 | DHFR | PLAU |  |
| G6PC1 | DAO | RORA |  |
| GCG | F9 | GPR35 |  |
| INSR | CDC7 | NR2F2 |  |
| IRS1 | CA12 | CES1 |  |
| PPARA | CA9 | APOBEC3G |  |
| SQSTM1 | CA2 | SIRT2 |  |
| TGFB1 | CA1 | GRM4 |  |
| ADIPOQ | HEXB | AHR |  |
| AGT | HEXA | HRH4 |  |
| CASP7 | ADORA1 | MIF |  |
| CYP7A1 | ACHE | CA9 |  |
| ESR1 | NUDT1 | ATP4A |  |
| FASN | ITGB3 | CYP1A2 |  |
| IGF1R |  | DRD5 |  |
| MMP9 |  | GALR3 |  |
| PCK1 |  | CHRM1 |  |
| RPS6KB1 |  | RPS6KB1 |  |
| SLC47A2 |  | CNR2 |  |
| SREBF1 |  | CHRM3 |  |
| XBP1 |  | AKR1B1 |  |
| AHR |  | CNR1 |  |
| HSPA5 |  | AR |  |
| MAPK1 |  | CA1 |  |
| MAPK3 |  | SIRT1 |  |
| NAMPT |  | HDAC4 |  |
| NR1I2 |  | ADRA2B |  |
| SLC22A1 |  | GRIN2B |  |
| SREBF2 |  | TAAR1 |  |
| CRP |  | RAC1 |  |
| CYP8B1 |  | METAP2 |  |
| DDIT3 |  | HRH2 |  |
| GCK |  | PTGS1 |  |
| GPT |  | DNMT1 |  |
| GSR |  | ESR2 |  |
| HSD3B |  | CXCR2 |  |
| IGF2 |  | CYP19A1 |  |
| IGF2R |  | ESR1 |  |
| IL10 |  | ANPEP |  |
| IL1RN |  | CSNK2A1 |  |
| KEAP1 |  | HRH1 |  |
| MRC1 |  | PRSS1 |  |
| MTOR |  | CA2 |  |
| MYC |  | ABCG2 |  |
| NFE2L1 |  | L3MBTL1 |  |
| NOS2 |  | MCL1 |  |
| NOS3 |  | CDC25B |  |
| RIPK1 |  | HTR6 |  |
| RPS6 |  | DUSP3 |  |
| SELENOP |  | S1PR4 |  |
| SLC10A1 |  | DPP7 |  |
| SLC15A1 |  | RPS6KA3 |  |
| TDO2 |  | CYP2C19 |  |
| TLR4 |  | SIGMAR1 |  |
| ACHE |  | LACTBL1 |  |
| AGER |  | DYRK1A |  |
| ATF6 |  | APP |  |
| BAD |  | GRIA2 |  |
| BCL2L1 |  | PLA2G1B |  |
| CCND2 |  | CTDSP1 |  |
| CCNE1 |  | PNP |  |
| CCNG2 |  | PTGS2 |  |
| CDKN1A |  | HTR3A |  |
| CXCL8 |  | ACE |  |
| CYBA |  | CTSL |  |
| CYP17A1 |  | DRD2 |  |
| CYP1B1 |  | DPP4 |  |
| CYP27A1 |  | CACNA1B |  |
| DDIT4 |  | TUBA1A |  |
| EIF2AK3 |  | PLIN1 |  |
| FN1 |  | F2 |  |
| GADD45A |  |  |  |
| GCLM |  |  |  |
| GCNT3 |  |  |  |
| H2AX |  |  |  |
| HAVCR1 |  |  |  |
| HIF1A |  |  |  |
| HMGCR |  |  |  |
| HNF4A |  |  |  |
| HRH2 |  |  |  |
| IL15RA |  |  |  |
| LDLR |  |  |  |
| ME1 |  |  |  |
| MMP2 |  |  |  |
| NFKB1 |  |  |  |
| NR3C1 |  |  |  |
| PFKFB1 |  |  |  |
| PIK3R1 |  |  |  |
| PRKCA |  |  |  |
| PRKCB |  |  |  |
| RIPK3 |  |  |  |
| RUNX2 |  |  |  |
| SLC2A2 |  |  |  |
| SOD1 |  |  |  |
| SP1 |  |  |  |
| TP53 |  |  |  |
| TXNIP |  |  |  |
| ABCB11 |  |  |  |
| ABCC9 |  |  |  |
| ABCG5 |  |  |  |
| ACE2 |  |  |  |
| ACLY |  |  |  |
| ACSL1 |  |  |  |
| ACTA2 |  |  |  |
| ANGPTL4 |  |  |  |
| APPL1 |  |  |  |
| ARG1 |  |  |  |
| ASNS |  |  |  |
| ATF4 |  |  |  |
| BCAT2 |  |  |  |
| BDNF |  |  |  |
| BECN1 |  |  |  |
| BTG2 |  |  |  |
| CARS1 |  |  |  |
| CASP1 |  |  |  |
| CASP8 |  |  |  |
| CCNB2 |  |  |  |
| CCND1 |  |  |  |
| CCNG1 |  |  |  |
| CDK4 |  |  |  |
| CHUK |  |  |  |
| COX1 |  |  |  |
| CPT1A |  |  |  |
| CREB3L2 |  |  |  |
| CXCL1 |  |  |  |
| CYCS |  |  |  |
| CYP1A2 |  |  |  |
| CYP3A11 |  |  |  |
| DEPTOR |  |  |  |
| DNMT1 |  |  |  |
| DUSP5 |  |  |  |
| EDA2R |  |  |  |
| EDN1 |  |  |  |
| EGR1 |  |  |  |
| EIF2S1 |  |  |  |
| EIF4E |  |  |  |
| EIF4EBP1 |  |  |  |
| ELOVL6 |  |  |  |
| ERN1 |  |  |  |
| ESRRA |  |  |  |
| FLVCR2 |  |  |  |
| FNDC5 |  |  |  |
| FOS |  |  |  |
| FOXO1 |  |  |  |
| GOT1 |  |  |  |
| HMGB1 |  |  |  |
| IARS1 |  |  |  |
| IFNG |  |  |  |
| IGFBP1 |  |  |  |
| IRS2 |  |  |  |
| LDHA |  |  |  |
| LEP |  |  |  |
| MAP1LC3B |  |  |  |
| MAPK8 |  |  |  |
| MFN2 |  |  |  |
| NAGLU |  |  |  |
| NARS1 |  |  |  |
| NFKBIA |  |  |  |
| NR1I3 |  |  |  |
| NRF1 |  |  |  |
| OLR1 |  |  |  |
| OSGIN1 |  |  |  |
| PDK1 |  |  |  |
| PDX1 |  |  |  |
| PHLDA3 |  |  |  |
| PIK3C3 |  |  |  |
| PIK3CA |  |  |  |
| PIK3CG |  |  |  |
| PKLR |  |  |  |
| PLD1 |  |  |  |
| POU5F1 |  |  |  |
| PPP1R15A |  |  |  |
| PTGS2 |  |  |  |
| RAB11A |  |  |  |
| RETN |  |  |  |
| RICTOR |  |  |  |
| RND3 |  |  |  |
| RPS6KA2 |  |  |  |
| RPS6KA5 |  |  |  |
| SCD1 |  |  |  |
| SESN2 |  |  |  |
| SIK1 |  |  |  |
| SIRT2 |  |  |  |
| SLC10A2 |  |  |  |
| SLC22A4 |  |  |  |
| SLC22A5 |  |  |  |
| SLC29A4 |  |  |  |
| SLC6A9 |  |  |  |
| SLCO1A4 |  |  |  |
| SLCO2B1 |  |  |  |
| SOD2 |  |  |  |
| SP7 |  |  |  |
| STAR |  |  |  |
| STK11 |  |  |  |
| TELO2 |  |  |  |
| TNFRSF10B |  |  |  |
| TNFRSF21 |  |  |  |
| TP53INP1 |  |  |  |
| UCP2 |  |  |  |
| ULK1 |  |  |  |
| VIM |  |  |  |
| XPC |  |  |  |
| XRCC1 |  |  |  |
| AACS |  |  |  |
| AARS1 |  |  |  |
| ABCB1B |  |  |  |
| ABCB4 |  |  |  |
| ABCB6 |  |  |  |
| ABCB9 |  |  |  |
| ABCC2 |  |  |  |
| ABCC3 |  |  |  |
| ABCC4 |  |  |  |
| ABCC5 |  |  |  |
| ABCG8 |  |  |  |
| ABHD4 |  |  |  |
| ABL1 |  |  |  |
| ABLIM3 |  |  |  |
| ACACB |  |  |  |
| ACAT2 |  |  |  |
| ACE |  |  |  |
| ACMSD |  |  |  |
| ACNAT2 |  |  |  |
| ACOT2 |  |  |  |
| ACOT3 |  |  |  |
| ACSM3 |  |  |  |
| ACTA1 |  |  |  |
| ACTN1 |  |  |  |
| ADGRE1 |  |  |  |
| ADH4 |  |  |  |
| ADIPOR1 |  |  |  |
| AEN |  |  |  |
| AGRN |  |  |  |
| AGRP |  |  |  |
| AGTR1A |  |  |  |
| AIFM1 |  |  |  |
| AIFM2 |  |  |  |
| AK3 |  |  |  |
| AK4 |  |  |  |
| AKR1B8-PS2 |  |  |  |
| AKT2 |  |  |  |
| AKT3 |  |  |  |
| ALAS2 |  |  |  |
| ALB |  |  |  |
| ALDH18A1 |  |  |  |
| ALDH1A1 |  |  |  |
| ALDOA |  |  |  |
| ALOX15 |  |  |  |
| ALPL |  |  |  |
| AMBP |  |  |  |
| ANGPT1 |  |  |  |
| ANGPTL8 |  |  |  |
| APAF1 |  |  |  |
| APEX1 |  |  |  |
| APOA5 |  |  |  |
| APOF |  |  |  |
| AQP7 |  |  |  |
| ARFGAP3 |  |  |  |
| ARFGEF2 |  |  |  |
| ARHGEF10 |  |  |  |
| AR.L |  |  |  |
| ARL1 |  |  |  |
| ARPC5L |  |  |  |
| ARRDC3 |  |  |  |
| ASL |  |  |  |
| ATF3 |  |  |  |
| ATF5 |  |  |  |
| ATM |  |  |  |
| ATOSA |  |  |  |
| ATP1A2 |  |  |  |
| ATP1A3 |  |  |  |
| ATP2A2 |  |  |  |
| ATP5MC2 |  |  |  |
| ATP6 |  |  |  |
| ATP6V0D2 |  |  |  |
| ATR |  |  |  |
| AURKB |  |  |  |
| AVEN |  |  |  |
| B2M |  |  |  |
| BAG1 |  |  |  |
| BCAR3 |  |  |  |
| BCL6 |  |  |  |
| BHLHE40 |  |  |  |
| BIRC5 |  |  |  |
| BMAL1 |  |  |  |
| BMF |  |  |  |
| BMP4 |  |  |  |
| BNIP3 |  |  |  |
| BPGM |  |  |  |
| BRD2 |  |  |  |
| BUB1B |  |  |  |
| CACNA1D |  |  |  |
| CANX |  |  |  |
| CASP14 |  |  |  |
| CASP4 |  |  |  |
| CASP5 |  |  |  |
| CASQ1 |  |  |  |
| CBY1 |  |  |  |
| CCDC38 |  |  |  |
| CCDC77 |  |  |  |
| CCDC90B |  |  |  |
| CCL24 |  |  |  |
| CCL27A |  |  |  |
| CCL7 |  |  |  |
| CCN2 |  |  |  |
| CCNB1 |  |  |  |
| CCNC |  |  |  |
| CCNF |  |  |  |
| CCR2 |  |  |  |
| CD14 |  |  |  |
| CD40LG |  |  |  |
| CD52 |  |  |  |
| CD63 |  |  |  |
| CD74 |  |  |  |
| CDC16 |  |  |  |
| CDC20 |  |  |  |
| CDC25A |  |  |  |
| CDC25B |  |  |  |
| CDC34 |  |  |  |
| CDC42 |  |  |  |
| CDH1 |  |  |  |
| CDIP1 |  |  |  |
| CDK1 |  |  |  |
| CDK2 |  |  |  |
| CDK5R1 |  |  |  |
| CDK6 |  |  |  |
| CDK7 |  |  |  |
| CDK8 |  |  |  |
| CDKN1B |  |  |  |
| CEBPA |  |  |  |
| CERS5 |  |  |  |
| CFD |  |  |  |
| CHEK1 |  |  |  |
| CHEK2 |  |  |  |
| CHIL3 |  |  |  |
| CISH |  |  |  |
| CKAP2 |  |  |  |
| CLDN2 |  |  |  |
| CLDN3 |  |  |  |
| CMBL |  |  |  |
| CNPPD1 |  |  |  |
| COL4A1 |  |  |  |
| CPTP |  |  |  |
| CREB1 |  |  |  |
| CREBRF |  |  |  |
| CRELD2 |  |  |  |
| CROT |  |  |  |
| CST3 |  |  |  |
| CTDP1 |  |  |  |
| CTDSP2 |  |  |  |
| CTH |  |  |  |
| CTNNB1 |  |  |  |
| CTSC |  |  |  |
| CTTN |  |  |  |
| CTTNBP2NL |  |  |  |
| CXCL13 |  |  |  |
| CXCL2 |  |  |  |
| CXCL9 |  |  |  |
| CYBB |  |  |  |
| CYP11A1 |  |  |  |
| CYP19A1 |  |  |  |
| CYP27B1 |  |  |  |
| CYP2B10 |  |  |  |
| CYP2B9 |  |  |  |
| CYP2C12 |  |  |  |
| CYP2E1 |  |  |  |
| CYP4A10 |  |  |  |
| CYP4A14 |  |  |  |
| CYP4F37 |  |  |  |
| CYP7B1 |  |  |  |
| DAPK1 |  |  |  |
| DCXR |  |  |  |
| DDHD1 |  |  |  |
| DDI2 |  |  |  |
| DDIT4L |  |  |  |
| DDX17 |  |  |  |
| DDX24 |  |  |  |
| DENR |  |  |  |
| DFFA |  |  |  |
| DGAT2 |  |  |  |
| DGKA |  |  |  |
| DHX37 |  |  |  |
| DHX9 |  |  |  |
| DIABLO |  |  |  |
| DLAT |  |  |  |
| DLGAP5 |  |  |  |
| DMBT1 |  |  |  |
| DMXL2 |  |  |  |
| DNAJB9 |  |  |  |
| DNM1L |  |  |  |
| DNMBP |  |  |  |
| DPEP1 |  |  |  |
| DPP9 |  |  |  |
| DUSP1 |  |  |  |
| DUSP22 |  |  |  |
| DUSP4 |  |  |  |
| E2F4 |  |  |  |
| ECH1 |  |  |  |
| ECI1 |  |  |  |
| EFNA1 |  |  |  |
| EIF1 |  |  |  |
| EIF2S2 |  |  |  |
| EIF3C |  |  |  |
| ELF4 |  |  |  |
| ELK4 |  |  |  |
| EMB |  |  |  |
| ENO2 |  |  |  |
| ENTPD7 |  |  |  |
| EP400 |  |  |  |
| EPRS1 |  |  |  |
| EQTN |  |  |  |
| ERBB3 |  |  |  |
| ERO1A |  |  |  |
| ESR2 |  |  |  |
| ESR2B |  |  |  |
| FAAP20 |  |  |  |
| FABP2 |  |  |  |
| FABP3 |  |  |  |
| FABP7 |  |  |  |
| FAM149A |  |  |  |
| FAM210A |  |  |  |
| FAM32A |  |  |  |
| FAS |  |  |  |
| FBP1 |  |  |  |
| FBXO22 |  |  |  |
| FBXO30 |  |  |  |
| FBXO32 |  |  |  |
| FECH |  |  |  |
| FGF1 |  |  |  |
| FGF15 |  |  |  |
| FGF21 |  |  |  |
| FGR |  |  |  |
| FICD |  |  |  |
| FIS1 |  |  |  |
| FKBP1A |  |  |  |
| FMO3 |  |  |  |
| FOXA2 |  |  |  |
| FOXO3 |  |  |  |
| FUT4 |  |  |  |
| G0S2 |  |  |  |
| G6PD |  |  |  |
| GAD1 |  |  |  |
| GARS1 |  |  |  |
| GBE1 |  |  |  |
| GBP1 |  |  |  |
| GBP1-PS1 |  |  |  |
| GCKR |  |  |  |
| GCLC |  |  |  |
| GDF15 |  |  |  |
| GFAP |  |  |  |
| GHRL |  |  |  |
| GHSR |  |  |  |
| GIMD1 |  |  |  |
| GLS |  |  |  |
| GMPPB |  |  |  |
| GMPR |  |  |  |
| GNMT |  |  |  |
| GOLPH3L |  |  |  |
| GOLPH3L-PS1 |  |  |  |
| GPAM |  |  |  |
| GPAT3 |  |  |  |
| GPD1L2 |  |  |  |
| GPHN |  |  |  |
| GPIHBP1 |  |  |  |
| GPNMB |  |  |  |
| GPR22 |  |  |  |
| GPRIN3 |  |  |  |
| GSK3A |  |  |  |
| GSN |  |  |  |
| GSPT1 |  |  |  |
| GSTA1 |  |  |  |
| GSTM2 |  |  |  |
| GSTM7 |  |  |  |
| GSTP1 |  |  |  |
| GSTT3 |  |  |  |
| GYPA |  |  |  |
| GYS1 |  |  |  |
| H1-2 |  |  |  |
| HAL |  |  |  |
| HAMP |  |  |  |
| HBB-B1 |  |  |  |
| HBB-BS |  |  |  |
| HEATR6 |  |  |  |
| HEMGN |  |  |  |
| HERC3 |  |  |  |
| HERPUD1 |  |  |  |
| HHEX |  |  |  |
| HIST2H3C2 |  |  |  |
| HNRNPK |  |  |  |
| HOOK2 |  |  |  |
| HRAS |  |  |  |
| HSC70-PS1 |  |  |  |
| HSC70-PS2 |  |  |  |
| HSPA9 |  |  |  |
| HSPH1 |  |  |  |
| HTATIP2 |  |  |  |
| HTR1A |  |  |  |
| HTR2A |  |  |  |
| HULC |  |  |  |
| HYKK |  |  |  |
| HYOU1 |  |  |  |
| ICAM1 |  |  |  |
| IDE |  |  |  |
| IDI1 |  |  |  |
| IFGGA4L |  |  |  |
| IFITM3 |  |  |  |
| IFITM7 |  |  |  |
| IFNA1 |  |  |  |
| IKBKB |  |  |  |
| IL17RB |  |  |  |
| IL33 |  |  |  |
| INMT |  |  |  |
| INSIG1 |  |  |  |
| INSIG2 |  |  |  |
| IRF2BP2 |  |  |  |
| IRF3 |  |  |  |
| IRF5 |  |  |  |
| IRF7 |  |  |  |
| ISCU |  |  |  |
| ITGB1 |  |  |  |
| ITGB8 |  |  |  |
| ITPR1 |  |  |  |
| ITPR2 |  |  |  |
| JKAMP |  |  |  |
| JUN |  |  |  |
| JUNB |  |  |  |
| JUND |  |  |  |
| KBTBD12 |  |  |  |
| KLF15 |  |  |  |
| KLF9 |  |  |  |
| KLHL33 |  |  |  |
| KRAS |  |  |  |
| KRT19 |  |  |  |
| KRT80 |  |  |  |
| LAMC2 |  |  |  |
| LARS1 |  |  |  |
| LCE1D |  |  |  |
| LCN2 |  |  |  |
| LCORL |  |  |  |
| LEAP2 |  |  |  |
| LGALS5 |  |  |  |
| LHB |  |  |  |
| LINGO3 |  |  |  |
| LMCD1 |  |  |  |
| LMNA |  |  |  |
| LOX |  |  |  |
| LPAR3 |  |  |  |
| LPIN2 |  |  |  |
| LSS |  |  |  |
| LTN1 |  |  |  |
| LURAP1L |  |  |  |
| LYST |  |  |  |
| MAD2L1 |  |  |  |
| MAFG |  |  |  |
| MAGOHB |  |  |  |
| MAN1A2 |  |  |  |
| MAP2K3 |  |  |  |
| MAP3K2 |  |  |  |
| MAP3K7 |  |  |  |
| MAP4K2 |  |  |  |
| MAPK10 |  |  |  |
| MAPK14 |  |  |  |
| MAPK9 |  |  |  |
| MCL1 |  |  |  |
| MCM10 |  |  |  |
| MCTP2 |  |  |  |
| MDM2 |  |  |  |
| MED29 |  |  |  |
| MEF2A |  |  |  |
| MEF2B |  |  |  |
| MEF2C |  |  |  |
| MEF2D |  |  |  |
| METTL1 |  |  |  |
| METTL22 |  |  |  |
| MFSD2A |  |  |  |
| MGAT2 |  |  |  |
| MGL2 |  |  |  |
| MID1IP1 |  |  |  |
| MIR1 |  |  |  |
| MIR125B |  |  |  |
| MIR146A |  |  |  |
| MIR152 |  |  |  |
| MIR155 |  |  |  |
| MIR15B |  |  |  |
| MIR190 |  |  |  |
| MIR1969 |  |  |  |
| MIR19A |  |  |  |
| MIR200A |  |  |  |
| MIR292 |  |  |  |
| MIR29B2 |  |  |  |
| MIR466F-2 |  |  |  |
| MIR6364 |  |  |  |
| MIR99B |  |  |  |
| MME |  |  |  |
| MMP10 |  |  |  |
| MMP14 |  |  |  |
| MMP19 |  |  |  |
| MOCOS |  |  |  |
| MPO |  |  |  |
| MSC |  |  |  |
| MSMO1 |  |  |  |
| MT1A |  |  |  |
| MT1F |  |  |  |
| MT1M |  |  |  |
| MT2A |  |  |  |
| MTDH |  |  |  |
| MUG2 |  |  |  |
| MVD |  |  |  |
| MX2 |  |  |  |
| MYD88 |  |  |  |
| MYH6 |  |  |  |
| MYH7 |  |  |  |
| MYZAP |  |  |  |
| NCAPD2 |  |  |  |
| NCF1 |  |  |  |
| NCOA1 |  |  |  |
| NEDD9 |  |  |  |
| NFKB2 |  |  |  |
| NIN |  |  |  |
| NINJ1 |  |  |  |
| NMUR1 |  |  |  |
| NOCT |  |  |  |
| NOD1 |  |  |  |
| NOP58 |  |  |  |
| NOS1 |  |  |  |
| NOX2 |  |  |  |
| NOX4 |  |  |  |
| NPHS1 |  |  |  |
| NPPA |  |  |  |
| NPY |  |  |  |
| NR0B2 |  |  |  |
| NR1D1 |  |  |  |
| NR4A1 |  |  |  |
| NR4A2 |  |  |  |
| NR4A3 |  |  |  |
| NRAS |  |  |  |
| NTPCR |  |  |  |
| NTRK2 |  |  |  |
| NUPR1 |  |  |  |
| OAT |  |  |  |
| OGA |  |  |  |
| OGG1 |  |  |  |
| OLR906 |  |  |  |
| ONECUT1 |  |  |  |
| OPA1 |  |  |  |
| ORAI3 |  |  |  |
| OTUB2 |  |  |  |
| OTUD1 |  |  |  |
| P2RX7 |  |  |  |
| P4HB |  |  |  |
| PANK1 |  |  |  |
| PBSN |  |  |  |
| PDE8A |  |  |  |
| PDHA1 |  |  |  |
| PDIA5 |  |  |  |
| PDK4 |  |  |  |
| PDLIM1 |  |  |  |
| PDLIM3 |  |  |  |
| PDPK1 |  |  |  |
| PDRG1 |  |  |  |
| PENK |  |  |  |
| PER1 |  |  |  |
| PGM2 |  |  |  |
| PHGDH |  |  |  |
| PHKG1 |  |  |  |
| PIAS2 |  |  |  |
| PIDD1 |  |  |  |
| PIK3CB |  |  |  |
| PILRA |  |  |  |
| PIR |  |  |  |
| PLA2G12A |  |  |  |
| PLAAT2 |  |  |  |
| PLAT |  |  |  |
| PLEKHB1 |  |  |  |
| PLIN2 |  |  |  |
| PLK2 |  |  |  |
| PLPPR1 |  |  |  |
| PLTP |  |  |  |
| PMEPA1 |  |  |  |
| PMM2 |  |  |  |
| PNPLA3 |  |  |  |
| PNPLA6 |  |  |  |
| PODXL |  |  |  |
| POLR3F |  |  |  |
| POMC |  |  |  |
| PON1 |  |  |  |
| PPAN |  |  |  |
| PPFIBP1 |  |  |  |
| PPM1D |  |  |  |
| PPM1K |  |  |  |
| PPP1R3C |  |  |  |
| PPP4R2 |  |  |  |
| PRC1 |  |  |  |
| PRDM1 |  |  |  |
| PRDX2 |  |  |  |
| PRKAB2 |  |  |  |
| PRKAG2 |  |  |  |
| PRKCZ |  |  |  |
| PRL |  |  |  |
| PSAT1 |  |  |  |
| PTEN |  |  |  |
| PTGFR |  |  |  |
| PTPN1 |  |  |  |
| PXMP4 |  |  |  |
| PYCR1 |  |  |  |
| PYGL |  |  |  |
| RAB33A |  |  |  |
| RAB5A |  |  |  |
| RAD51C |  |  |  |
| RAET1B |  |  |  |
| RAF1 |  |  |  |
| RBP4 |  |  |  |
| RBP7 |  |  |  |
| RD3L |  |  |  |
| REG3G |  |  |  |
| RETNLA |  |  |  |
| RFFL |  |  |  |
| RGD1309362 |  |  |  |
| RGS2 |  |  |  |
| RHD |  |  |  |
| RHOA |  |  |  |
| RHOBTB1 |  |  |  |
| RIPK2 |  |  |  |
| RLF |  |  |  |
| RNASEL |  |  |  |
| RND1 |  |  |  |
| RNF121 |  |  |  |
| RO60 |  |  |  |
| RRM2 |  |  |  |
| RRM2B |  |  |  |
| RSL1D1 |  |  |  |
| RTN3 |  |  |  |
| RUNX1 |  |  |  |
| RXRA |  |  |  |
| SALL1 |  |  |  |
| SAMT2 |  |  |  |
| SARS1 |  |  |  |
| SAT1 |  |  |  |
| SBK1 |  |  |  |
| SCAMP1 |  |  |  |
| SCD |  |  |  |
| SCD3 |  |  |  |
| SDF2L1 |  |  |  |
| SDS |  |  |  |
| SEC23B |  |  |  |
| SEC62 |  |  |  |
| SELP |  |  |  |
| SEPHS2 |  |  |  |
| SERPINC1 |  |  |  |
| SERPINE1 |  |  |  |
| SERTAD1 |  |  |  |
| SESN1 |  |  |  |
| SETX |  |  |  |
| SEZ6 |  |  |  |
| SFN |  |  |  |
| SGK1 |  |  |  |
| SGK2 |  |  |  |
| SH3BP5 |  |  |  |
| SIAH2 |  |  |  |
| SIRT4 |  |  |  |
| SIRT5 |  |  |  |
| SIRT7 |  |  |  |
| SIVA1 |  |  |  |
| SKP2 |  |  |  |
| SLC12A8 |  |  |  |
| SLC16A10 |  |  |  |
| SLC16A6 |  |  |  |
| SLC19A2 |  |  |  |
| SLC1A4 |  |  |  |
| SLC25A20 |  |  |  |
| SLC25A37 |  |  |  |
| SLC25A47 |  |  |  |
| SLC2A5 |  |  |  |
| SLC3A2 |  |  |  |
| SLC41A3 |  |  |  |
| SLC46A3 |  |  |  |
| SLC4A1 |  |  |  |
| SLC6A6 |  |  |  |
| SLC7A1 |  |  |  |
| SLC7A11 |  |  |  |
| SLC7A5 |  |  |  |
| SLCO1A2 |  |  |  |
| SMAGP |  |  |  |
| SNAI2 |  |  |  |
| SNORA17 |  |  |  |
| SNORA30 |  |  |  |
| SNORD13 |  |  |  |
| SON |  |  |  |
| SORL1 |  |  |  |
| SP3 |  |  |  |
| SP4 |  |  |  |
| SPSB1 |  |  |  |
| SRD5A3 |  |  |  |
| SRM |  |  |  |
| SRR |  |  |  |
| SST |  |  |  |
| ST3GAL2 |  |  |  |
| STRBP |  |  |  |
| SULF2 |  |  |  |
| SULT2A2 |  |  |  |
| SULT4A1 |  |  |  |
| TAF15 |  |  |  |
| TALDO1 |  |  |  |
| TANC2 |  |  |  |
| TAOK1 |  |  |  |
| TBC1D4 |  |  |  |
| TBCCD1 |  |  |  |
| TBX3 |  |  |  |
| TCEAL7 |  |  |  |
| TCP11L2 |  |  |  |
| TFAM |  |  |  |
| TFE3 |  |  |  |
| TFRC |  |  |  |
| TH |  |  |  |
| THRSP |  |  |  |
| TIMM17B |  |  |  |
| TIMP3 |  |  |  |
| TIPARP |  |  |  |
| TKFC |  |  |  |
| TLR1 |  |  |  |
| TLR2 |  |  |  |
| TLR3 |  |  |  |
| TLR5 |  |  |  |
| TLR6 |  |  |  |
| TLR7 |  |  |  |
| TLR8 |  |  |  |
| TMEM140 |  |  |  |
| TMEM71 |  |  |  |
| TMOD4 |  |  |  |
| TMPRSS2 |  |  |  |
| TNFAIP8 |  |  |  |
| TNFRSF10A |  |  |  |
| TNFRSF1A |  |  |  |
| TNFRSF25 |  |  |  |
| TNFRSF9 |  |  |  |
| TNFSF12 |  |  |  |
| TPCN1 |  |  |  |
| TRADD |  |  |  |
| TRAF2 |  |  |  |
| TRAF3 |  |  |  |
| TRAF4 |  |  |  |
| TRAFD1 |  |  |  |
| TRIM21 |  |  |  |
| TRIM39 |  |  |  |
| TRPC1 |  |  |  |
| TSKU |  |  |  |
| TSPAN12 |  |  |  |
| TXNRD1 |  |  |  |
| UAP1L1 |  |  |  |
| UBE2B |  |  |  |
| UBE2G2 |  |  |  |
| UGT2A3 |  |  |  |
| ULK2 |  |  |  |
| UNKL |  |  |  |
| USP3 |  |  |  |
| VARS1 |  |  |  |
| VDR |  |  |  |
| VEGFA |  |  |  |
| VEGFB |  |  |  |
| VGLL4 |  |  |  |
| VNN1 |  |  |  |
| VPS53 |  |  |  |
| VSNL1 |  |  |  |
| VWF |  |  |  |
| WARS1 |  |  |  |
| WWC2 |  |  |  |
| YARS1 |  |  |  |
| YPEL2 |  |  |  |
| ZBTB16 |  |  |  |
| ZBTB21 |  |  |  |
| ZFP239 |  |  |  |
| ZFP354A |  |  |  |
| ZFP36 |  |  |  |
| ZFP46 |  |  |  |
| ZFP57 |  |  |  |
| ZFP715 |  |  |  |
| ZFP791 |  |  |  |
| ZFP871 |  |  |  |
| ZFP982 |  |  |  |

Supplementary Table 2. Univariate survival cox regression in the non-small cell lung cancer patients from the TCGA cohort

| ID | HR | HR.95L | HR.95H | p-value |
| --- | --- | --- | --- | --- |
| CCL14 | 0.680142995 | 0.469436532 | 0.985424999 | 0.04159222 |
| ARRDC5 | 0.82898084 | 0.688672509 | 0.997875222 | 0.047429314 |
| KRTAP2-2 | 0.00735955 | 0.000125549 | 0.431410455 | 0.018044353 |
| ZDHHC11 | 0.96577597 | 0.940167063 | 0.99208243 | 0.011094869 |
| PNMA8C | 0.474241633 | 0.234385277 | 0.95955313 | 0.038006202 |
| SCN1A | 0.808776854 | 0.670619947 | 0.975395978 | 0.02637673 |
| AL441992.2 | 0.041772926 | 0.001955262 | 0.892451795 | 0.042072523 |
| ATXN7 | 0.971167456 | 0.943337631 | 0.999818301 | 0.048585217 |
| IRX5 | 0.991022787 | 0.983933443 | 0.998163211 | 0.013821307 |
| CERKL | 0.933719374 | 0.876282281 | 0.994921259 | 0.034246932 |
| IL2 | 0.550765371 | 0.327101925 | 0.927363828 | 0.024856338 |
| ZNF77 | 0.972953031 | 0.948600369 | 0.997930879 | 0.033996142 |
| GATA1 | 0.707695886 | 0.506355101 | 0.989095332 | 0.042954048 |
| AMIGO1 | 0.97510828 | 0.951924456 | 0.998856738 | 0.040059186 |
| ZNF556 | 0.928794365 | 0.863229783 | 0.999338751 | 0.047965547 |
| ZNF709 | 0.209369889 | 0.061897797 | 0.708195654 | 0.011906588 |
| NDUFA7 | 0.776793085 | 0.622312951 | 0.969620663 | 0.025571242 |
| ZNF540 | 0.832693294 | 0.737787449 | 0.939807424 | 0.003022269 |
| OGT | 0.996131639 | 0.992780928 | 0.999493658 | 0.024159973 |
| CBX7 | 0.978185754 | 0.959972216 | 0.996744857 | 0.021450302 |
| CD1E | 0.98154833 | 0.96447412 | 0.998924807 | 0.037514811 |
| FNIP2 | 0.991462013 | 0.983595897 | 0.999391037 | 0.03487135 |
| ZNF441 | 0.929814211 | 0.877529429 | 0.985214215 | 0.013722894 |
| ANKRD44 | 0.95522053 | 0.92750804 | 0.983761025 | 0.002289045 |
| CCDC190 | 0.991585226 | 0.983375272 | 0.999863723 | 0.046360943 |
| COX14 | 0.99673487 | 0.993680546 | 0.999798582 | 0.03674304 |
| COL4A3 | 0.976948119 | 0.960007295 | 0.994187891 | 0.008973002 |
| BORCS8 | 0.958770277 | 0.924612472 | 0.994189967 | 0.022918889 |
| KLRB1 | 0.980674597 | 0.963601571 | 0.998050122 | 0.029422581 |
| DHRS4L2 | 0.982500741 | 0.965679104 | 0.999615403 | 0.045110634 |
| CCDC177 | 0.916825076 | 0.847755998 | 0.991521408 | 0.029777643 |
| SLC38A4 | 0.978940353 | 0.960039189 | 0.998213641 | 0.03237866 |
| CD302 | 0.96511804 | 0.94027506 | 0.990617395 | 0.00761977 |
| DNASE2B | 0.928251188 | 0.867718606 | 0.993006561 | 0.030469021 |
| MTUS1 | 0.994201461 | 0.989700048 | 0.998723348 | 0.012014807 |
| ZNF248 | 0.962494305 | 0.927713593 | 0.998578973 | 0.041781387 |
| HLA-DMB | 0.995068658 | 0.991520716 | 0.998629296 | 0.006675562 |
| NECAB3 | 0.995236835 | 0.990713503 | 0.999780819 | 0.039948776 |
| FGFBP2 | 0.999465259 | 0.998950264 | 0.999980519 | 0.041947015 |
| STK33 | 0.954612487 | 0.918760847 | 0.99186312 | 0.017393307 |
| MUSK | 0.645792888 | 0.457223833 | 0.912131922 | 0.013065035 |
| GALNT11 | 0.991965384 | 0.984006562 | 0.999988579 | 0.049676073 |
| ATP13A4 | 0.993489339 | 0.988532239 | 0.998471297 | 0.010484868 |
| LIFR | 0.991784032 | 0.984950347 | 0.998665129 | 0.019355373 |
| NRXN3 | 0.957102773 | 0.918964532 | 0.996823802 | 0.034575096 |
| DNAJC28 | 0.905955887 | 0.823917877 | 0.996162471 | 0.041414493 |
| BTBD9 | 0.988018345 | 0.979354709 | 0.996758622 | 0.007308261 |
| GLB1L3 | 0.985166545 | 0.974117005 | 0.996341421 | 0.00940778 |
| ROM1 | 0.961168158 | 0.929573088 | 0.993837106 | 0.02020757 |
| GPR31 | 0.501833433 | 0.261157711 | 0.964309242 | 0.038543895 |
| ITIH4 | 0.559479687 | 0.314928877 | 0.99393083 | 0.047621913 |
| CYP17A1 | 0.483881261 | 0.249124763 | 0.939854681 | 0.03210565 |
| MOK | 0.953102422 | 0.917864134 | 0.989693565 | 0.012456336 |
| ZNF747 | 0.969943398 | 0.944532976 | 0.996037429 | 0.024252823 |
| SMAD9 | 0.972359224 | 0.948772224 | 0.996532609 | 0.025273776 |
| AC010547.4 | 0.00013174 | 2.36E-08 | 0.735113978 | 0.042369006 |
| BTK | 0.981095223 | 0.964565933 | 0.997907767 | 0.027696575 |
| ABHD14B | 0.995837517 | 0.991832412 | 0.999858794 | 0.042494558 |
| ZNF20 | 0.373998383 | 0.189612925 | 0.737685946 | 0.004542376 |
| SCART1 | 0.904912887 | 0.830684761 | 0.985773871 | 0.022132836 |
| ACOXL | 0.930471656 | 0.88379322 | 0.979615462 | 0.006065015 |
| AQP6 | 0.869315504 | 0.757124723 | 0.998130721 | 0.046977471 |
| ENPP5 | 0.991930726 | 0.985192592 | 0.998714945 | 0.019821121 |
| SEPTIN1 | 0.975082428 | 0.95136139 | 0.99939492 | 0.044629214 |
| AC012651.1 | 0.143005204 | 0.027503068 | 0.743571171 | 0.020765702 |
| WDR83 | 0.932713199 | 0.874639277 | 0.994643088 | 0.033693142 |
| ZNF44 | 0.934624903 | 0.897978384 | 0.972766967 | 0.000923376 |
| CFAP91 | 0.917092734 | 0.84220613 | 0.998638044 | 0.046445485 |
| VMAC | 0.972042484 | 0.945457095 | 0.99937543 | 0.045057107 |
| CCDC181 | 0.937459176 | 0.88358875 | 0.994613961 | 0.032450232 |
| RPS6KA5 | 0.898399779 | 0.817869868 | 0.986858905 | 0.02534917 |
| GGA2 | 0.994205304 | 0.988646494 | 0.999795369 | 0.042204027 |
| SETDB2 | 0.960665451 | 0.926084904 | 0.996537255 | 0.031919807 |
| LDHD | 0.99101166 | 0.983488759 | 0.998592104 | 0.020214416 |
| PZP | 0.927189491 | 0.861558685 | 0.997819845 | 0.043567166 |
| ZSCAN31 | 0.990949442 | 0.982636412 | 0.999332799 | 0.034409207 |
| NDNF | 0.998207863 | 0.996527413 | 0.999891148 | 0.03692484 |
| CARMIL3 | 0.902933725 | 0.843353104 | 0.966723557 | 0.003371687 |
| CES4A | 0.969651882 | 0.941972988 | 0.998144091 | 0.03700733 |
| CA5B | 0.932771927 | 0.878152677 | 0.99078838 | 0.02378644 |
| SENP8 | 0.884563215 | 0.785440905 | 0.996194718 | 0.043089901 |
| GMPR | 0.989194544 | 0.979776042 | 0.998703586 | 0.026032355 |
| HPGDS | 0.971885087 | 0.94505338 | 0.999478592 | 0.045882091 |
| CABLES1 | 0.987855291 | 0.977004804 | 0.998826283 | 0.030130395 |
| TREML1 | 0.882393539 | 0.796195778 | 0.977923245 | 0.017050384 |
| EPHX1 | 0.999749952 | 0.999531992 | 0.999967959 | 0.024577044 |
| HLA-DMA | 0.998724208 | 0.997763067 | 0.999686276 | 0.009358061 |
| SETD4 | 0.974605218 | 0.951456584 | 0.99831705 | 0.035967183 |
| NRTN | 0.981670961 | 0.963678829 | 0.999999012 | 0.049987759 |
| TSLP | 0.951926511 | 0.918207618 | 0.986883646 | 0.007417248 |
| DMRTC1 | 7.10E-05 | 5.75E-08 | 0.087724242 | 0.008539675 |
| TRIP6 | 0.998553034 | 0.997148717 | 0.999959328 | 0.04373652 |
| HLF | 0.984648761 | 0.973668502 | 0.995752847 | 0.006854144 |
| CYP2U1 | 0.955740094 | 0.922752543 | 0.98990692 | 0.011536373 |
| FDX2 | 0.174549476 | 0.040838001 | 0.746058052 | 0.018510778 |
| ADAMTS8 | 0.973613362 | 0.949154304 | 0.998702713 | 0.039401994 |
| ACSBG1 | 0.683930662 | 0.493713424 | 0.947434539 | 0.022330221 |
| UPRT | 0.975890665 | 0.953125959 | 0.999199089 | 0.042713993 |
| GGTLC1 | 0.996679762 | 0.994028434 | 0.999338161 | 0.014400729 |
| JAML | 0.981638681 | 0.964836246 | 0.998733727 | 0.035395355 |
| SORCS2 | 0.989725578 | 0.981086546 | 0.998440681 | 0.020952547 |
| KLHDC8B | 0.991516369 | 0.986966008 | 0.99608771 | 0.000283169 |
| HHATL | 0.808159463 | 0.658102395 | 0.992431759 | 0.042107456 |
| BEST4 | 0.922322868 | 0.862360855 | 0.986454182 | 0.018392713 |
| DEXI | 0.919400361 | 0.860635099 | 0.98217819 | 0.012646638 |
| PAPLN | 0.982376438 | 0.96760681 | 0.99737151 | 0.021420536 |
| MPP7 | 0.992030615 | 0.985420369 | 0.998685204 | 0.018993266 |
| RNFT1 | 0.980271116 | 0.962862683 | 0.99799429 | 0.029289002 |
| ZNF589 | 0.972227793 | 0.955431621 | 0.989319236 | 0.00153669 |
| CFTR | 0.981703676 | 0.966745339 | 0.996893461 | 0.018417005 |
| SCN4A | 0.891268441 | 0.803306925 | 0.988861679 | 0.029913209 |
| SLC4A8 | 0.925842197 | 0.870037582 | 0.985226146 | 0.015131466 |
| INAFM2 | 0.985482777 | 0.975855624 | 0.995204906 | 0.003504729 |
| STIMATE-MUSTN1 | 0.295772216 | 0.111247091 | 0.786368464 | 0.014619157 |
| SLC47A1 | 0.982928106 | 0.970341215 | 0.995678269 | 0.008828821 |
| TRDMT1 | 0.861405201 | 0.758534411 | 0.978227104 | 0.021492447 |
| TMEM150A | 0.99382647 | 0.988303929 | 0.999379871 | 0.029394863 |
| RFTN2 | 0.927453618 | 0.860385623 | 0.999749636 | 0.049240639 |
| BCDIN3D | 0.929980409 | 0.865518416 | 0.999243396 | 0.047634443 |
| SCN4B | 0.975894977 | 0.952929499 | 0.999413921 | 0.044620548 |
| CX3CR1 | 0.969695439 | 0.944257012 | 0.99581918 | 0.023277187 |
| ABCA13 | 0.988995582 | 0.978332337 | 0.99977505 | 0.045431226 |
| DMD | 0.967493762 | 0.937097605 | 0.998875864 | 0.042455831 |
| RNF180 | 0.95884205 | 0.922830319 | 0.996259071 | 0.031408652 |
| TSPOAP1 | 0.974929345 | 0.952114941 | 0.998290424 | 0.035589286 |
| NUDT14 | 0.995407597 | 0.990861071 | 0.999974984 | 0.048761136 |
| SIX1 | 0.989967643 | 0.983029727 | 0.996954524 | 0.004954337 |
| AMPH | 0.954831393 | 0.912603474 | 0.999013279 | 0.045205224 |
| CPED1 | 0.96179965 | 0.934182966 | 0.990232749 | 0.008785747 |
| GPD1L | 0.993625749 | 0.989721917 | 0.997544979 | 0.001453683 |
| XCR1 | 0.913034377 | 0.846617375 | 0.984661783 | 0.018221064 |
| SMARCE1 | 0.894209779 | 0.805320195 | 0.992910811 | 0.036336274 |
| CXCL17 | 0.999785846 | 0.999611069 | 0.999960653 | 0.016346753 |
| CLHC1 | 0.932751828 | 0.871294312 | 0.998544304 | 0.045300446 |
| UQCR10 | 0.996990698 | 0.994140608 | 0.999848959 | 0.039076543 |
| DRAM1 | 0.998633671 | 0.997316921 | 0.99995216 | 0.042251831 |
| RAB44 | 0.583866861 | 0.417139109 | 0.817234594 | 0.001710404 |
| ERC2 | 0.885694348 | 0.790886642 | 0.991867149 | 0.035611857 |
| SLC26A5 | 0.882127659 | 0.792823147 | 0.981491534 | 0.02127814 |
| CD74 | 0.999924836 | 0.999862817 | 0.999986859 | 0.017539467 |
| RNF175 | 0.906344523 | 0.844111242 | 0.973166041 | 0.006740264 |
| KCNJ11 | 0.983307695 | 0.967100893 | 0.999786093 | 0.047122949 |
| ZNF254 | 0.980105445 | 0.963401162 | 0.997099361 | 0.021953953 |
| ANKRD45 | 0.914407963 | 0.84340919 | 0.991383462 | 0.030020711 |
| ZNF396 | 0.918353297 | 0.844243154 | 0.998969046 | 0.04725684 |
| STIMATE | 0.548435784 | 0.310869035 | 0.967551527 | 0.038093305 |
| KCNJ15 | 0.992559814 | 0.985619519 | 0.999548979 | 0.036981131 |
| STING1 | 0.997375233 | 0.994908116 | 0.999848468 | 0.037535581 |
| TESPA1 | 0.932865615 | 0.880042876 | 0.988858929 | 0.019456122 |
| IL33 | 0.99559408 | 0.991904795 | 0.999297087 | 0.019743433 |
| GLIPR1L2 | 0.831921767 | 0.694893349 | 0.995971292 | 0.045078115 |
| RORA | 0.969221224 | 0.941398006 | 0.997866762 | 0.035407758 |
| TTLL2 | 0.718981451 | 0.531251063 | 0.973050904 | 0.032605309 |
| ZNF563 | 0.917199977 | 0.852978638 | 0.986256584 | 0.019616102 |
| NIBAN1 | 0.995430295 | 0.99093809 | 0.999942864 | 0.047175253 |
| C1QL2 | 0.988970531 | 0.979701215 | 0.998327547 | 0.020979811 |
| GRIA1 | 0.881302207 | 0.792154482 | 0.980482465 | 0.020221389 |
| TCTA | 0.991121922 | 0.985777347 | 0.996495473 | 0.001226998 |
| ZNF502 | 0.960143723 | 0.93423377 | 0.986772261 | 0.003568297 |
| ZNF831 | 0.845963387 | 0.728766867 | 0.982006844 | 0.027905832 |
| FAM214A | 0.98275527 | 0.966238032 | 0.99955486 | 0.044278137 |
| FYB2 | 0.943968527 | 0.896811844 | 0.993604829 | 0.027430473 |
| NKIRAS1 | 0.94813932 | 0.905982976 | 0.992257244 | 0.021737124 |
| GDF15 | 0.999328055 | 0.998674671 | 0.999981867 | 0.043977485 |
| ZNF626 | 0.972349022 | 0.94584154 | 0.999599384 | 0.046769925 |
| DAPK2 | 0.969938193 | 0.941068955 | 0.999693055 | 0.047717341 |
| CRYGN | 0.914158033 | 0.845195135 | 0.988747893 | 0.024914458 |
| CLECL1 | 0.898767034 | 0.833085118 | 0.96962743 | 0.005841339 |
| TMA7 | 0.989321646 | 0.97900401 | 0.999748018 | 0.044741298 |
| SNAI3 | 0.938925637 | 0.891239297 | 0.989163465 | 0.017804062 |
| POU2AF1 | 0.992720695 | 0.986054679 | 0.999431774 | 0.033560841 |
| HLA-DPB1 | 0.999066132 | 0.998283025 | 0.999849854 | 0.019528726 |
| ACSM5 | 0.860189576 | 0.741870669 | 0.99737884 | 0.046072687 |
| SPTLC2 | 0.993833758 | 0.987922601 | 0.999780284 | 0.042137613 |
| C6 | 0.962928101 | 0.930065298 | 0.996952075 | 0.032985129 |
| CD200R1 | 0.879787029 | 0.818371521 | 0.945811525 | 0.00052255 |
| NPRL2 | 0.977408818 | 0.961206408 | 0.993884342 | 0.007378986 |
| BDH2 | 0.977362463 | 0.955986264 | 0.999216641 | 0.042415511 |
| ZNF10 | 0.960436161 | 0.922555661 | 0.99987205 | 0.049275922 |
| MS4A2 | 0.95536086 | 0.919380269 | 0.992749576 | 0.019727937 |
| CACNA2D2 | 0.995932328 | 0.992058769 | 0.999821011 | 0.040365014 |
| ZNF57 | 0.689726352 | 0.493514378 | 0.963948493 | 0.029633916 |
| PGM5 | 0.964253822 | 0.935844088 | 0.993526 | 0.017049537 |
| CSAD | 0.985101806 | 0.971909442 | 0.998473237 | 0.029103417 |
| FRS3 | 0.979422708 | 0.959589512 | 0.999665826 | 0.046372692 |
| KCNK16 | 0.73252251 | 0.543725769 | 0.986874742 | 0.040673126 |
| GPLD1 | 0.903548643 | 0.839765839 | 0.972175947 | 0.006618457 |
| C12orf42 | 0.765102116 | 0.587878466 | 0.995752154 | 0.04641179 |
| TTC23L | 0.74588939 | 0.615449969 | 0.903974345 | 0.002795981 |
| MAGEH1 | 0.996211806 | 0.992622854 | 0.999813734 | 0.039290887 |
| OPHN1 | 0.971251844 | 0.945375098 | 0.997836887 | 0.034248499 |
| PLPPR1 | 0.979507828 | 0.965452852 | 0.993767415 | 0.004987984 |
| STK32A | 0.9819232 | 0.968548505 | 0.995482587 | 0.009133493 |
| EFHC2 | 0.969995667 | 0.94454975 | 0.996127091 | 0.024699831 |
| TMEM163 | 0.993528825 | 0.987346553 | 0.999749808 | 0.04149663 |
| TLR2 | 0.994450335 | 0.989201604 | 0.999726916 | 0.039291781 |
| IVD | 0.992533267 | 0.986662628 | 0.998438837 | 0.013280713 |
| PDIK1L | 0.984159573 | 0.970344987 | 0.998170834 | 0.026842253 |
| CLEC2D | 0.963551499 | 0.935195567 | 0.992767207 | 0.014839409 |
| MADCAM1 | 0.823474187 | 0.688774386 | 0.984516485 | 0.033073307 |
| TMEM243 | 0.991604419 | 0.98385541 | 0.99941446 | 0.035179166 |
| CRY2 | 0.99111363 | 0.982974111 | 0.999320548 | 0.033879112 |
| SLC38A6 | 0.966719174 | 0.937503056 | 0.996845775 | 0.030638647 |
| HAPLN4 | 8.81E-25 | 1.41E-48 | 0.552793945 | 0.047572595 |
| NPC2 | 0.999678399 | 0.9993688 | 0.999988094 | 0.041820737 |
| C12orf76 | 0.967888493 | 0.944430093 | 0.991929569 | 0.009126592 |
| FUCA1 | 0.99890866 | 0.997852948 | 0.99996549 | 0.042977242 |
| BCL6 | 0.995592307 | 0.991361445 | 0.999841224 | 0.042048133 |
| AC055839.2 | 0.975589449 | 0.953588629 | 0.998097863 | 0.033706477 |
| C3orf62 | 0.942345935 | 0.895663582 | 0.991461391 | 0.021976975 |
| SH2B1 | 0.989110826 | 0.980338805 | 0.997961339 | 0.015997774 |
| RTL5 | 0.975649514 | 0.957424038 | 0.99422193 | 0.010399124 |
| HLA-DPA1 | 0.999442456 | 0.998917323 | 0.999967864 | 0.037542921 |
| ADHFE1 | 0.903355551 | 0.829929313 | 0.98327802 | 0.018781725 |
| PHKG2 | 0.974214475 | 0.956651332 | 0.992100059 | 0.004886198 |
| AZIN2 | 0.940971064 | 0.89104927 | 0.993689769 | 0.028701473 |
| ATAD3C | 0.98041954 | 0.966838302 | 0.994191556 | 0.005461637 |
| CXorf21 | 0.974715859 | 0.952526658 | 0.99742196 | 0.029281865 |
| PDZD9 | 0.547895778 | 0.316035555 | 0.949860795 | 0.032097304 |
| CYP4X1 | 0.996217558 | 0.992901847 | 0.999544342 | 0.025886806 |
| C5orf38 | 0.992818733 | 0.986325142 | 0.999355076 | 0.031345943 |
| AL669918.1 | 6.17E-14 | 1.20E-25 | 0.031603801 | 0.027030062 |
| PARP15 | 0.972285443 | 0.946960125 | 0.998288057 | 0.036869533 |
| NR3C2 | 0.97945293 | 0.961670949 | 0.997563711 | 0.026357598 |
| CYP4B1 | 0.998914527 | 0.997999499 | 0.999830394 | 0.020193908 |
| RSBN1L | 0.986201125 | 0.973435354 | 0.999134307 | 0.0365955 |
| HMGN3 | 0.998629419 | 0.997662467 | 0.999597309 | 0.005522722 |
| LRP2BP | 0.921667412 | 0.854329708 | 0.99431263 | 0.035090575 |
| LRRC3C | 0.573475678 | 0.332512207 | 0.989059488 | 0.045551802 |
| FITM1 | 0.735030275 | 0.569458633 | 0.948742322 | 0.018077062 |
| MYLIP | 0.993285219 | 0.989437373 | 0.997148029 | 0.000668533 |
| ZNF555 | 0.878280898 | 0.780362926 | 0.988485372 | 0.031397939 |
| RNF130 | 0.984358141 | 0.970352954 | 0.998565466 | 0.031059302 |
| AKR1A1 | 0.997680725 | 0.99566084 | 0.999704708 | 0.024730662 |
| RORB | 0.906359767 | 0.829990283 | 0.989756199 | 0.028579718 |
| TP53INP1 | 0.995453124 | 0.99093013 | 0.999996761 | 0.049837296 |
| CD5 | 0.984934532 | 0.972862587 | 0.997156275 | 0.015841012 |
| SLC6A18 | 0.475759051 | 0.245285899 | 0.922787146 | 0.027970581 |
| GGT6 | 0.993576239 | 0.98761607 | 0.999572377 | 0.035791151 |
| KHDRBS2 | 0.952151267 | 0.915630463 | 0.990128738 | 0.014006442 |
| CHADL | 0.959882419 | 0.924015497 | 0.997141563 | 0.035092465 |
| SLC34A2 | 0.999847828 | 0.999740568 | 0.999955101 | 0.005431635 |
| GRM6 | 0.535401829 | 0.286814838 | 0.999443126 | 0.049795897 |
| GCSAML | 0.848885164 | 0.721043536 | 0.999393221 | 0.049154135 |
| ANKRD29 | 0.986046492 | 0.97415933 | 0.998078706 | 0.023162718 |
| SPRYD4 | 0.950414206 | 0.911419633 | 0.99107714 | 0.017346549 |
| EPHX2 | 0.986625374 | 0.974046226 | 0.999366974 | 0.039716452 |
| AC068896.1 | 0.498061359 | 0.276288813 | 0.897847127 | 0.020429537 |
| ADCY9 | 0.982615416 | 0.969676001 | 0.995727497 | 0.009513264 |
| C1QTNF7 | 0.938571354 | 0.888405484 | 0.991569956 | 0.02369561 |
| ABCC8 | 0.801549868 | 0.663822366 | 0.967852582 | 0.021467785 |
| LRIG1 | 0.991391289 | 0.985811376 | 0.997002785 | 0.002679428 |
| LUZP2 | 0.840532847 | 0.7537067 | 0.937361267 | 0.001791615 |
| C11orf16 | 0.968745831 | 0.943968745 | 0.994173261 | 0.016304349 |
| CPXM2 | 0.997691483 | 0.995392556 | 0.999995719 | 0.049576346 |
| FGD3 | 0.978448905 | 0.958094415 | 0.99923582 | 0.042230867 |
| PRDM16 | 0.972508229 | 0.950535494 | 0.99498889 | 0.016810956 |
| SEC14L6 | 0.982740289 | 0.966545779 | 0.999206139 | 0.040010455 |
| LIPT1 | 0.941602391 | 0.89877145 | 0.986474439 | 0.011299936 |
| SLC5A11 | 0.928723368 | 0.864088066 | 0.998193503 | 0.044527436 |
| PRICKLE4 | 0.34207715 | 0.134383331 | 0.870768537 | 0.024433609 |
| FHAD1 | 0.934791948 | 0.874377624 | 0.999380545 | 0.047912719 |
| CLIC6 | 0.998488383 | 0.997394836 | 0.99958313 | 0.006815299 |
| TCEA3 | 0.997095605 | 0.99426213 | 0.999937156 | 0.045150672 |
| DNASE2 | 0.997412208 | 0.994860385 | 0.999970578 | 0.047426076 |
| CLEC9A | 0.802776467 | 0.651876708 | 0.988607274 | 0.038657119 |
| SLC16A11 | 0.971679963 | 0.946643842 | 0.997378221 | 0.030999699 |
| USP4 | 0.98734307 | 0.976091844 | 0.998723988 | 0.029382341 |
| FAM189A2 | 0.967588336 | 0.938951073 | 0.997099013 | 0.031595324 |
| SNX30 | 0.98756777 | 0.979062877 | 0.996146543 | 0.004584531 |
| POU6F1 | 0.952595766 | 0.910176561 | 0.996991938 | 0.036654945 |
| MS4A4E | 0.844896556 | 0.728881518 | 0.979377544 | 0.025320516 |
| KLRG1 | 0.900473456 | 0.829695982 | 0.977288626 | 0.012073092 |
| GAPT | 0.941481949 | 0.894988373 | 0.990390812 | 0.019614457 |
| C19orf18 | 0.936474809 | 0.882150024 | 0.994145036 | 0.031353784 |
| NAPSA | 0.999799713 | 0.999620469 | 0.99997899 | 0.028551012 |
| CCDC88C | 0.989040207 | 0.979004246 | 0.999179049 | 0.034192627 |
| DENND1C | 0.984927343 | 0.973630298 | 0.996355468 | 0.009871775 |
| ELAPOR1 | 0.996668945 | 0.994257557 | 0.999086181 | 0.00694062 |
| PXMP4 | 0.990542728 | 0.982875092 | 0.998270181 | 0.016546598 |
| ZNF506 | 0.962768867 | 0.933415586 | 0.993045225 | 0.016317171 |
| SGF29 | 0.981831099 | 0.967380769 | 0.996497282 | 0.015359342 |
| HLA-DRB1 | 0.999922592 | 0.999850385 | 0.999994804 | 0.035641458 |
| ZNF655 | 0.989318037 | 0.981162004 | 0.997541868 | 0.01100096 |
| TLR5 | 0.966373879 | 0.939640322 | 0.993868029 | 0.016862489 |
| HLA-DQA1 | 0.99875874 | 0.997684999 | 0.999833635 | 0.023628013 |
| TMEM50B | 0.994028275 | 0.988968157 | 0.999114284 | 0.021433129 |
| ZDHHC11B | 0.97356783 | 0.956650866 | 0.990783947 | 0.002742529 |
| NDUFA11 | 0.950788783 | 0.909252303 | 0.994222734 | 0.026815767 |
| IKZF4 | 0.968797757 | 0.94010323 | 0.998368119 | 0.038787855 |
| ZFP3 | 0.967238578 | 0.937184757 | 0.998256172 | 0.038608172 |
| TTC39B | 0.977458505 | 0.957220666 | 0.998124219 | 0.032691457 |
| PELI1 | 0.996966751 | 0.994320546 | 0.999619999 | 0.025074958 |
| SH3BP5 | 0.971715595 | 0.945471254 | 0.998688424 | 0.039983989 |
| PPP1R13B | 0.98515627 | 0.975219419 | 0.995194372 | 0.003836666 |
| RAP1GAP | 0.997806301 | 0.995758292 | 0.999858523 | 0.036176997 |
| CIITA | 0.981773459 | 0.965599523 | 0.998218311 | 0.029978736 |
| ESYT3 | 0.968489403 | 0.946386243 | 0.991108789 | 0.006564528 |
| CDKL2 | 0.9883453 | 0.977363902 | 0.999450082 | 0.039738563 |
| NUPR1 | 0.941850049 | 0.905168116 | 0.980018518 | 0.003118701 |
| SUSD3 | 0.986057076 | 0.974710351 | 0.997535889 | 0.017418137 |
| P2RY13 | 0.979879241 | 0.962610065 | 0.997458226 | 0.02505871 |
| PET100 | 0.894432157 | 0.80599707 | 0.992570462 | 0.0356976 |
| FMC1 | 0.76633645 | 0.642104477 | 0.914604361 | 0.003186724 |
| TTC28 | 0.96767066 | 0.941877131 | 0.994170551 | 0.017120825 |
| TFAP2E | 0.96799915 | 0.938676932 | 0.99823733 | 0.03823026 |
| ZC3H12D | 0.901195054 | 0.833919358 | 0.973898157 | 0.008586051 |
| ATG16L2 | 0.978151858 | 0.961360663 | 0.995236329 | 0.012403039 |
| SCUBE3 | 0.991290524 | 0.982980825 | 0.999670469 | 0.04168002 |
| SELENOK | 0.994979278 | 0.990230284 | 0.999751048 | 0.039211342 |
| CERS4 | 0.994695636 | 0.990992855 | 0.998412253 | 0.0051893 |
| PLEKHB1 | 0.99382922 | 0.988433328 | 0.999254568 | 0.025852045 |
| ENTPD3 | 0.994518436 | 0.989189706 | 0.999875871 | 0.044936237 |
| ZNF25 | 0.962930847 | 0.935332533 | 0.99134349 | 0.01089793 |
| GDAP1 | 0.939094419 | 0.889561801 | 0.991385115 | 0.023031163 |
| HLA-DOB | 0.977892547 | 0.960303899 | 0.995803343 | 0.015774043 |
| PPM1M | 0.983204423 | 0.972292043 | 0.994239278 | 0.00293434 |
| SLC9A9 | 0.987844119 | 0.978537432 | 0.99723932 | 0.011329458 |
| PRKCE | 0.962829194 | 0.928067403 | 0.998893027 | 0.043487286 |
| GSTA3 | 0.93681156 | 0.88679901 | 0.989644655 | 0.019709692 |
| HLA-DRA | 0.999955024 | 0.999914056 | 0.999995993 | 0.031425131 |
| HLA-DQB1 | 0.999159714 | 0.998323994 | 0.999996134 | 0.048950814 |
| CPEB3 | 0.916397955 | 0.846559287 | 0.991998109 | 0.030880692 |
| ECHDC2 | 0.988245742 | 0.977713596 | 0.998891343 | 0.030550656 |
| MICU3 | 0.895091215 | 0.833508378 | 0.96122403 | 0.002308547 |
| SLC25A42 | 0.967623143 | 0.945511341 | 0.990252053 | 0.005262664 |
| PXK | 0.972498911 | 0.94870119 | 0.996893586 | 0.02737729 |
| SULT1A3 | 0.139251638 | 0.023732148 | 0.817078094 | 0.028981684 |
| CPA3 | 0.997448698 | 0.994924783 | 0.999979015 | 0.048132317 |
| CCL17 | 0.995187668 | 0.990410944 | 0.99998743 | 0.049404478 |
| RPS27AP5 | 0.927570356 | 0.861593837 | 0.998599024 | 0.045802959 |
| EXPH5 | 0.97399312 | 0.955529939 | 0.992813054 | 0.006962202 |
| COL21A1 | 0.986378048 | 0.973525137 | 0.999400649 | 0.04040804 |
| ALK | 0.885408345 | 0.803484416 | 0.975685306 | 0.014015468 |
| JPH1 | 0.983969309 | 0.973961362 | 0.994080093 | 0.001946348 |
| CATSPERE | 0.764891676 | 0.636169596 | 0.919659286 | 0.004361129 |
| ZNF490 | 0.851868713 | 0.749068016 | 0.968777585 | 0.014549804 |
| SUOX | 0.98886409 | 0.978045601 | 0.999802246 | 0.046020948 |
| TPPP | 0.985873109 | 0.976131382 | 0.995712059 | 0.004983637 |
| TMEM168 | 0.984038512 | 0.970437667 | 0.997829975 | 0.02345831 |
| PTCHD4 | 0.93463348 | 0.880818843 | 0.991735985 | 0.025468147 |
| NRL | 0.799768075 | 0.652174928 | 0.9807629 | 0.031826709 |
| MOAP1 | 0.994019139 | 0.988400677 | 0.999669539 | 0.038056232 |
| PGPEP1 | 0.98539868 | 0.972315251 | 0.998658158 | 0.031016737 |
| CIRBP | 0.996623615 | 0.994147197 | 0.999106203 | 0.007712335 |
| NEIL1 | 0.973185554 | 0.950976444 | 0.995913335 | 0.021019503 |
| MAPK13 | 0.996576292 | 0.99331108 | 0.999852236 | 0.040538646 |
| FAM13B | 0.978633437 | 0.958141064 | 0.999564094 | 0.045463036 |
| C10orf143 | 0.860964036 | 0.749091342 | 0.989544306 | 0.035033929 |
| FAIM2 | 0.956667721 | 0.925973776 | 0.9883791 | 0.007756202 |
| CLEC18B | 0.782235333 | 0.615801352 | 0.993651791 | 0.044205273 |
| GAB3 | 0.955856807 | 0.91855024 | 0.994678566 | 0.02623985 |
| SCGB3A1 | 0.999912682 | 0.999843292 | 0.999982076 | 0.013656412 |
| AQP5 | 0.99923591 | 0.99854519 | 0.999927109 | 0.030267234 |
| PLAAT1 | 0.991378352 | 0.982998454 | 0.999829687 | 0.045576863 |
| ZNF512 | 0.985380076 | 0.972509539 | 0.998420946 | 0.028124399 |
| GLS2 | 0.474748396 | 0.260398592 | 0.865542467 | 0.015048517 |
| MCTP2 | 0.978730087 | 0.95933776 | 0.998514416 | 0.03524221 |
| CCDC66 | 0.935778039 | 0.881973589 | 0.992864809 | 0.028022284 |
| SUSD4 | 0.995545784 | 0.991148107 | 0.999962974 | 0.048114422 |
| ZFP2 | 0.878913847 | 0.787282203 | 0.981210484 | 0.021582814 |
| TRAF3IP3 | 0.952530883 | 0.914505893 | 0.992136947 | 0.019296331 |
| PDE6C | 0.653063025 | 0.44524517 | 0.957879711 | 0.029246086 |
| SLAMF1 | 0.955953527 | 0.920358267 | 0.992925449 | 0.019982815 |
| DAAM2 | 0.978657874 | 0.95944143 | 0.998259201 | 0.032993348 |
| ZNF493 | 0.9433298 | 0.896381855 | 0.992736641 | 0.02510086 |
| SPATA6L | 0.920612741 | 0.852569492 | 0.994086495 | 0.034741652 |
| SLC25A23 | 0.994940663 | 0.990407032 | 0.999495046 | 0.029501519 |
| MCOLN2 | 0.958426488 | 0.931501335 | 0.986129916 | 0.003492952 |
| FCRL6 | 0.941681084 | 0.892385775 | 0.993699461 | 0.028498234 |
| ITPKB | 0.995339898 | 0.990716724 | 0.999984646 | 0.049249184 |
| GDPD1 | 0.975112729 | 0.951653087 | 0.999150685 | 0.042524695 |
| STX10 | 0.99648642 | 0.993223938 | 0.999759619 | 0.035409062 |
| SFTA3 | 0.996733214 | 0.993843816 | 0.999631012 | 0.027165854 |
| INPP5J | 0.960014621 | 0.93338417 | 0.987404868 | 0.004468344 |
| NWD1 | 0.965521666 | 0.934325373 | 0.997759576 | 0.036276734 |
| MTURN | 0.991200934 | 0.982494865 | 0.999984148 | 0.049589759 |
| ATP8A2 | 0.93283723 | 0.8830762 | 0.985402275 | 0.012928728 |
| CCR2 | 0.970032615 | 0.946390097 | 0.994265766 | 0.015659568 |
| FAM184A | 0.961090742 | 0.934294233 | 0.988655802 | 0.005945958 |
| LRRC4 | 0.996357881 | 0.993297023 | 0.999428171 | 0.020107613 |
| GNG7 | 0.950917799 | 0.924002495 | 0.97861712 | 0.00059166 |
| GSTA4 | 0.998083623 | 0.996436143 | 0.999733828 | 0.022858203 |
| ZNF483 | 0.922704343 | 0.853192026 | 0.997880053 | 0.044107343 |
| SCN7A | 0.973409171 | 0.951166876 | 0.996171585 | 0.022300785 |
| ABAT | 0.974494347 | 0.957159762 | 0.992142869 | 0.00478219 |
| WFIKKN2 | 0.615344273 | 0.41604424 | 0.910116131 | 0.015032122 |
| BTLA | 0.920412457 | 0.850150676 | 0.996481112 | 0.04066096 |
| RMDN2 | 0.952971708 | 0.921466808 | 0.985553758 | 0.004979979 |
| GUCY1A1 | 0.984238903 | 0.96879038 | 0.999933771 | 0.049048559 |
| GCGR | 0.936659885 | 0.878673376 | 0.998473113 | 0.044767992 |
| ATP5MG | 0.987693935 | 0.97585101 | 0.999680587 | 0.04423365 |
| DNAH12 | 0.926031883 | 0.863602634 | 0.9929741 | 0.030931163 |
| PCP2 | 0.932558626 | 0.888931478 | 0.978326916 | 0.004285982 |
| NGF | 0.98883847 | 0.978835514 | 0.998943649 | 0.030487048 |
| ANKDD1B | 0.954898315 | 0.91327726 | 0.99841618 | 0.042389594 |
| THSD7B | 0.955185366 | 0.91874522 | 0.993070837 | 0.020869688 |
| ETV1 | 0.990349267 | 0.982083646 | 0.998684456 | 0.023340788 |
| PIGR | 0.999795397 | 0.999629566 | 0.999961254 | 0.015615508 |
| MGP | 0.998968613 | 0.998103063 | 0.999834913 | 0.019633835 |
| ICAM5 | 0.984491682 | 0.970981902 | 0.99818943 | 0.026621896 |
| AC008770.2 | 0.038450777 | 0.002497315 | 0.592020748 | 0.019504663 |
| CD40LG | 0.942938641 | 0.903200637 | 0.984424994 | 0.007483426 |
| ABCC4 | 0.990376678 | 0.981479541 | 0.999354467 | 0.035710141 |
| ZNF791 | 0.971617393 | 0.950934858 | 0.992749767 | 0.008721018 |
| ANKHD1-EIF4EBP3 | 0.557671507 | 0.322658906 | 0.96385844 | 0.03645467 |
| AC006254.1 | 0.638115317 | 0.432702812 | 0.941041162 | 0.023416934 |
| CCDC153 | 0.955946863 | 0.920171814 | 0.993112799 | 0.020607754 |
| ADGRF5 | 0.998674768 | 0.997598539 | 0.999752159 | 0.015929252 |
| CAPN3 | 0.914039679 | 0.83975877 | 0.994891109 | 0.03767215 |
| IRX6 | 0.988003241 | 0.978464764 | 0.997634702 | 0.014752501 |
| PRRT3 | 0.970287422 | 0.942708957 | 0.998672682 | 0.040340546 |
| RALGPS1 | 0.961316328 | 0.927259418 | 0.996624101 | 0.032055932 |
| FBXO9 | 0.985931009 | 0.974356807 | 0.997642699 | 0.018688939 |
| SUSD2 | 0.998933384 | 0.998044265 | 0.999823296 | 0.018827301 |
| ZNF682 | 0.953950867 | 0.923692784 | 0.985200137 | 0.004148777 |
| SEC14L3 | 0.845486792 | 0.724501955 | 0.986674929 | 0.033153501 |
| FYCO1 | 0.989384585 | 0.980030856 | 0.99882759 | 0.027664451 |
| PRKCD | 0.996169201 | 0.992687338 | 0.999663277 | 0.031675381 |
| FAM200A | 0.981042195 | 0.965045963 | 0.997303574 | 0.022497075 |
| LYRM9 | 0.93017539 | 0.865713771 | 0.999436864 | 0.048229971 |
| NFIX | 0.996793645 | 0.993784991 | 0.999811408 | 0.037319301 |
| MAGI3 | 0.989091742 | 0.979031869 | 0.999254984 | 0.035478534 |
| ANOS1 | 0.987683181 | 0.979802247 | 0.995627505 | 0.002428981 |
| DMTF1 | 0.986682587 | 0.974423322 | 0.999096086 | 0.035576921 |
| TMEM215 | 0.728697084 | 0.537299285 | 0.98827498 | 0.041767491 |
| AL365205.1 | 0.833712654 | 0.695915237 | 0.998795188 | 0.048490698 |
| CLEC18A | 0.258180149 | 0.072389985 | 0.920804019 | 0.036875686 |
| CRYM | 0.994886928 | 0.991061798 | 0.998726821 | 0.009103075 |
| AC144573.1 | 0.000338318 | 1.26E-07 | 0.910543104 | 0.047342797 |
| CISH | 0.992667945 | 0.986882687 | 0.998487118 | 0.013600351 |
| GPR174 | 0.946765711 | 0.902479584 | 0.99322503 | 0.025215064 |
| ITGA8 | 0.983574019 | 0.96745955 | 0.999956898 | 0.049405209 |
| NOTCH2NLR | 0.302663702 | 0.124078017 | 0.738288045 | 0.008617234 |
| ABCC6 | 0.984890498 | 0.970685603 | 0.999303267 | 0.039976305 |
| CENPC | 0.968280479 | 0.937986626 | 0.999552723 | 0.046861513 |
| CLNK | 0.818396202 | 0.687836213 | 0.973738124 | 0.023815796 |
| MERTK | 0.993451162 | 0.987090083 | 0.999853234 | 0.044989951 |
| CAV3 | 0.517396489 | 0.283290272 | 0.944964064 | 0.032019772 |
| IRX2 | 0.996450707 | 0.993085564 | 0.999827253 | 0.039392874 |
| NEUROD1 | 0.91860464 | 0.844291773 | 0.999458376 | 0.048546687 |
| GPRIN2 | 0.989239229 | 0.980589038 | 0.997965726 | 0.015761298 |
| LCA5 | 0.954986444 | 0.924521635 | 0.986455128 | 0.005362632 |
| KCNS3 | 0.996988714 | 0.994268427 | 0.999716444 | 0.030510294 |
| CLEC3B | 0.988719879 | 0.980048504 | 0.997467978 | 0.011601378 |
| AMPD1 | 0.902609774 | 0.822030227 | 0.991088135 | 0.031746568 |
| CACNB4 | 0.620695871 | 0.439711071 | 0.8761739 | 0.00669696 |
| CTF1 | 0.991465314 | 0.984057225 | 0.998929173 | 0.02509385 |
| SMARCD3 | 0.988087679 | 0.97765887 | 0.998627732 | 0.026854985 |
| AL139353.1 | 0.498814657 | 0.257350763 | 0.966836308 | 0.039413191 |
| SCML4 | 0.852971658 | 0.737660429 | 0.98630836 | 0.031872899 |
| HLA-DRB5 | 0.999851388 | 0.999708947 | 0.999993848 | 0.040895278 |
| ANKRD65 | 0.99472875 | 0.991277817 | 0.998191697 | 0.002875629 |
| SNX20 | 0.976059624 | 0.953369607 | 0.999289659 | 0.043468882 |
| ERO1B | 0.990852813 | 0.983933794 | 0.997820486 | 0.01016285 |
| ZNF394 | 0.955060021 | 0.921356132 | 0.989996823 | 0.012127331 |
| KMT2E | 0.989494468 | 0.980555122 | 0.998515311 | 0.02255759 |
